# Transcriptomic Analysis of *Musca domestica* to Reveal Key Genes of the Prophenoloxidase-Activating System

**DOI:** 10.1534/g3.115.016899

**Published:** 2015-07-07

**Authors:** Dianxiang Li, Yongli Liang, Xianwei Wang, Lei Wang, Mei Qi, Yang Yu, Yuanyuan Luan

**Affiliations:** * Biotechnology Department, The School of Biological Sciences and Biotechnology, University of Jinan, Jinan 250022, People’s Republic of China; † Shandong Academy of Medical Sciences, Jinan 250002, People’s Republic of China; ‡ School of Life Sciences, Shandong University, Jinan 250100, People’s Republic of China; § School of Medicine and Life Science, Shandong Academy of Medical Sciences, University of Jinan, Jinan 250022, People’s Republic of China

**Keywords:** prophenoloxidase-activating system, gene identification, transcriptomic analysis, *Musca domestica*, genetics of immunity

## Abstract

The proPO system regulates melanization in arthropods. However, the genes that are involved in the proPO system in housefly *Musca domestica* remain unclear. Thus, this study analyzed the combined transcriptome obtained from *M. domestica* larvae, pupae, and adults that were either normal or bacteria-challenged by an *Escherichia coli* and *Staphylococcus aureus* mixture. A total of 54,821,138 clean reads (4.93 Gb) were yielded by Illumina sequencing, which were *de novo* assembled into 89,842 unigenes. Of the 89,842 unigenes, based on a similarity search with known genes in other insects, 24 putative genes related to the proPO system were identified. Eight of the identified genes encoded for peptidoglycan recognition receptors, two encoded for prophenoloxidases, three encoded for prophenoloxidase-activating enzymes, and 11 encoded for serine proteinase inhibitors. The expression levels of these identified genes were investigated by qRT-PCR assay, which were consistent with expected activation process of the proPO system, and their activation functions were confirmed by the measurement of phenoloxidase activity in bacteria-infected larvae after proPO antibody blockage, suggesting these candidate genes might have potentially different roles in the activation of proPO system. Collectively, this study has provided the comprehensive transcriptomic data of an insect and some fundamental basis toward achieving understanding of the activation mechanisms and immune functions of the proPO system in *M. domestica*.

The housefly *Musca domestica* is a worldwide insect vector that can transport numerous pathogenic organisms, including parasites, viruses, bacteria, and even antibiotic-resistant bacteria (Tan *et al.* 1997; Sasaki *et al.* 2000; Davari *et al.* 2012; Schuster *et al.* 2013; Wei *et al.* 2013). These pathogens can cause more than 100 serious diseases in human and animals, such as salmonellosis, typhoid fever, cholera, infantile diarrhea, and amoebic dysentery (Scott *et al.* 2014). In addition to the public health threat, the housefly can suppress milk and egg production in livestock and poultry farming, as well as reduce food conversion. Confusingly, this species can resist infections and maintain its growing prosperity even living in an environment full of pathogens. However, until recently, little has been known about the molecular mechanism of housefly immune response to these pathogens (Li *et al.* 2013; Liu *et al.* 2012; Scott *et al.* 2014; Tang *et al.* 2014).

Insects rely on their innate immune system as a defense against pathogens because they lack an acquired immune system (Kingsolver and Hardy 2012). The prophenoloxidase-activating system (proPO system) can produce melanin within a few minutes after pathogen invasion and participate in host innate immune responses, including killing, eliminating, or inhibiting invading pathogens (Rao *et al.* 2010; Qian *et al.* 2013). The proPO system has been extensively investigated in various insect species, such as *Drosophila* (An *et al.* 2013), *Anopheles* (An *et al.* 2011a), *Tenebrio molitor* (Tindwa *et al.* 2013), *Manduca sexta* (An *et al.* 2011b; Wang *et al.* 2011, 2014), and *Bombyx mori* (Chen *et al.* 2014), and the activation cascade has also been preliminarily summarized as follows (Cerenius and Söderhäll 2004; Cerenius *et al.* 2008, 2010). Generally, invaders are recognized by pattern recognition proteins of the host, such as peptidoglycan recognition proteins (PGRPs) or β-1,3-glucan recognition proteins, and then a cascade reaction of serine proteases is initiated in which many serine proteases are involved, including prophenoloxidase (proPO), prophenoloxidase-activating enzymes (PAPs), serine protease inhibitors (Serpins), and serine protease homologs (SPHs). Once activated, proPO is released into the plasma and converted into phenoloxidase (PO) via restrictive proteolysis. PO is the last and most important component of the proPO system, which oxidizes phenol into benzoquinone that is then polymerized into insoluble melanin by nonenzymatic reactions. The melanin is deposited at the injury site or on the invading pathogens to induce the blackening and healing of wound (Tindwa *et al.* 2013).

Studies on the contribution of melanization to the survival of dipterans obtained variable results. Two reports on *Drosophila* (Leclerc *et al.* 2006) and *Anopheles* (Schnitger *et al.* 2007) revealed that the proPO system exhibits no bactericidal activity, but recent work has demonstrated that *Drosophila* requires proPO activation to survive microbial infections (Binggeli *et al.* 2014). Therefore, it is crucial to identify the genes related to the proPO system in other dipterans, such as *M. domestica* for elucidating the activation mechanisms and immune functions of this system.

Although there were a few reports about PO (Sun *et al.* 2008), PO inhibitors (Tsukamoto *et al.* 1992), and proPO sequence (AAR84669) in the past, current knowledge of this proPO system is limited compared with antimicrobial peptides and pattern recognition proteins in *M. domestica* (Wang *et al.* 2006; Fu *et al.* 2009; Ai *et al.* 2013; Sun *et al.* 2014). In addition, no report is available on the PGRPs, PAPs, and Serpins of *M. domestica*.

We previously constructed a suppression subtractive hybridization (SSH) cDNA library of *M. domestica* larvae and identified a few cDNA segments of PAP and proPO (Li *et al.* 2010). However, the SSH library yielded limited genomic resources (Qiu *et al.* 2012); therefore, the main components and the activation mechanisms of the proPO system in *M. domestica* remain unclear.

For the past few years, the high-throughput technology RNA-Seq has been used to produce millions of short cDNA reads and cost-effectively assemble transcriptomes for nonmodel organisms with unknown genomes (Grabherr *et al.* 2011). This technology has opened a door for numerous and substantial studies on gene discovery. RNA-Seq has also been used for *M. domestica* transcriptomic analyses using insecticide-resistant adult flies (Li *et al.* 2013) or 3-d-old bacteria-infected larvae (Liu *et al.* 2012) or different developmental stages, including normal eggs, pupae, adults, and bacteria-challenged and nonchallenged larvae (Tang *et al.* 2014). Nevertheless, the key genes involved in the proPO system in *M. domestica* have yet to be identified.

In this study, we mixed 15 kinds of RNA samples from normal and bacteria-challenged (*E. coli* and *Staphylococcus aureus* mixture) larvae, pupae, and adults and conducted the RNA-Seq using Illumina paired-end sequencing. An enormous amount of transcriptomic data were generated. Furthermore, many candidate genes involved in the proPO system were identified and characterized through transcriptomic analysis and real-time quantitative reverse-transcriptase polymerase chain reaction (qRT-PCR) assay as well as the measurement of PO activity.

## Materials and Methods

### Housefly culture

Housefly (*Musca domestica*) strains were kindly gifted by Jinan City Center for Disease Control and Prevention in China. Larvae, pupae, and adult flies were maintained in our laboratory at 25° to 30°, 50 70% humidity, and 12 hr light/12 hr dark cycle. The larvae were reared on an artificial medium of bran and milk power until pupation, and adult flies were fed water with sugar. The challenged samples were collected by lightly piercing the postabdomen of healthy third instar larvae, pupae, and adult flies with 1-mL disposable syringe needles previously immersed into a mixed culture of *E. coli* and *S. aureus* (1.5 × 10^8^ CFU/mL). The samples were maintained on fresh medium until RNA extraction.

### RNA isolation and Illumina sequencing

Total RNA samples were extracted from housefly larvae, pupae, and adults that were either normal or bacteria-challenged (at 12, 24, and 48 hr) using TRIzol reagent (Invitrogen, USA) following the manufacturer’s instructions. After DNase I treatment, RNA integrity was examined using 2100 Bioanalyzer (Agilent Technologies, USA). Then, the qualified RNA samples were mixed equally and used to isolate poly(A) mRNA using magnetic beads with Oligo(dT). The mRNAs were fragmented (200 nt to 700 nt) with RNA fragmentation reagents. The first-strand cDNAs and then the second-strand cDNAs were synthesized using a random hexamer primer with the short mRNA fragments as templates according to the cDNA kit (BD Biosciences Clontech) manufacturer’s instructions. The cDNA fragments were purified and resolved with an EB buffer and then end-repaired with an adapter primer following the manufacturer’s protocol (Illumina). The suitable cDNA fragments were selected as templates by agarose gel electrophoresis to create the final cDNA library by PCR amplification. After quantification and qualification, the cDNA library was sequenced using Illumina HiSeq 2000 in the Beijing Genomics Institute (Shenzhen, China).

### Transcriptome *de novo* assembly

Transcriptome *de novo* assembly was performed using the assembling program Trinity (Grabherr *et al.* 2011). Before assembly, the raw reads first were filtered to obtain high-quality clean reads by removing dirty reads that contain adapter sequences, or unknown nucleotides (N) larger than 5%, or low-quality bases (base quality ≤10) more than 20%. Then, Trinity combined the clean reads to form contigs with a certain length of overlap and further connected the contigs to obtain unigenes that cannot be extended on either end. The unigenes were divided into two classes through gene family clustering. The first class contained clusters with the prefix CL and the cluster ID, in which several unigenes demonstrated more than 70% similarity. The second class contained singletons with the prefix unigene. The contigs and the unigenes were longer than 200 and 300 nt, respectively.

### Annotation of unigenes

On the basis of sequence similarity with known genes, each assembled unigene underwent protein functional annotation, pathway annotation, Cluster of Orthologous Groups (COG) functional annotation, and Gene Ontology (GO) functional annotation. Briefly, the unigenes were first aligned by BLASTx search against the protein databases of NCBI nonredundant sequence database (Nr), Swiss-Prot, Kyoto Encyclopedia of Genes and Genomes (KEGG), and COG. None of the BLASTx hits were aligned to NCBI nonredundant nucleotide database (Nt) by BLASTn. All alignments were performed using a cutoff E-value ≤10^−5^ to determine the homology of sequences with known genes. The best alignment results were used to decide the sequence direction and the protein coding region prediction (CDSs) of unigenes. When the different databases yielded contradictory results, *a priori* order of Nr, Swiss-Prot, KEGG, and COG was followed. If a unigene was not aligned to any of the mentioned databases, then ESTScan (Iseli *et al.* 1999) was used to decide the sequence direction and to predict the coding regions. After the prediction of unigene CDSs, proteins with the highest ranks in BLAST results were analyzed to decide the coding region sequences of the unigenes. With the help of the KEGG database, the complicated biological behavior of the genes were further studied through pathway annotation. All unigenes were aligned to the COG database to predict and classify their possible functions. On the basis of Nr annotation, GO annotations of the unigenes were obtained using Blast2GO (Conesa *et al.* 2005). WEGO (Ye *et al.* 2006) was used for the GO functional classification of all unigenes to understand the distribution of gene functions at the macro level.

### Identification of key genes related to the proPO system

Available key genes such as PGRPs, PAPs, Serpins, SPHs, and proPOs in other insect species were used as references to examine this *M. domestica* transcriptome. The reference species included *Drosophila melanogaster*, *M. sexta*, *B. mori*, *T. molitor*, *Holotrichia diomphalia*, and *Rhipicephalus microplus*. The potential candidate unigenes in the *M. domestica* transcriptome were confirmed by sequence analysis, protein domain prediction, and phylogenetic analysis. The sequence analyses of the hypothetical genes were performed using BLAST (http://blast.ncbi.nlm.nih.gov/Blast.cgi) and DNAMAN 8.0. Meanwhile, the protein domains of putative genes were determined by SMART (http://www.smart.embl-heidelberg.de/). In addition, complete or partial amino acid sequences, signal peptides, and open reading frames from the available nucleotides of the unigenes were deduced using ExPASy (http://www.expasy.org). The sequence alignments and the phylogenetic analyses of putative genes were implemented using GeneDoc (Nicholas *et al.* 1997) and MEGA 5.0 (Tamura *et al.* 2011), respectively. The cDNA segments of defined genes were cloned using PCR primers designed according to the representative unigenes and subsequently sequenced by Sangon Company (Shanghai, China).

### Real-time RT-PCR analysis

Real-time RT-PCR (qRT-PCR) was performed using iQSYBR Green Supermix (Bio-Rad) and CFX96 Real-Time System (Bio-Rad) to investigate the expression profiles of selected genes that might be related to the proPO system in *M. domestica*. Total RNAs were isolated from third instar larvae that were normal or bacteria-challenged by *E. coli* or *S. aureus* (at 4, 6, 12, and 24 hr), and then were synthesized into cDNAs using the aforementioned methods. The total volume of qRT-PCR mixture was 10 µL each including 5 µL of Supermix, 2 µL of each primer (1 µM), and 1 µL of diluted cDNA. The qRT-PCR program included an initial 95° for 5 min, 40 cycles of 95° for 10 sec, and 60° for 1 min, and then a melt period from 65° to 95°. Every sample was tested in triplicate and the qRT-PCR data were normalized twice. The first normalization was completed using actin internal reference to get a ∆Ct value (Ct_target_ − Ct_actin_), and then the second was done by the comparison between ∆Ct value of the experimental sample and that of the control sample (∆∆Ct = ∆Ct_target_ − ∆Ct_control_). Results were expressed as the mean ± SD from three independent repeats using the 2^−ΔΔCt^ data with Graph-Pad Prism (Swift 1997). The statistical significance (***P < 0.001, **P < 0.01, *P < 0.05) was detected by *t*-test. The primers are listed in Supporting Information, Table S1.

### Measurement of phenoloxidase activity

The third instar larvae were divided into two clusters, one by sample and a separate one by control. The control larvae were all normal, whereas sample larvae were divided into four groups again, including group 1 in which the larvae were lightly pierced in the postabdomen with 1-mL syringe needles previously immersed into 1×PBS, group 2 with larvae injected with 5 µL anti-mdproPO serum (previously made in our laboratory) for 30 min, group 3 with larvae challenged with 1-mL syringe needles previously immersed into the mixture of *S. aureus* and *E. coli* (1.5 × 10^8^ CFU/mL) for 30 min, and group 4 with larvae that first were injected with 5 µL anti-mdproPO serum for 30 min and then challenged by the mixture of *S. aureus* and *E. coli* for 30 min. The hemolymph of larvae (N = 30) in each group was collected in 200 µL anticoagulation buffer (NaCl 110.5 mmol/L, KCl 2 mmol/L, NaHCO_3_ 2.4 mmol/L, NaH_2_PO_4_ 0.083 mmol/L, EDTA 20 mmol/L; pH 7.0) on ice to reduce spontaneous activation of the proPO cascade *in vitro*, respectively. Phenoloxidase activity was assayed with L-DOPA (BBI) as a substrate using a previously published 96-well microplates method by our laboratory (Guo *et al.* 2014). Briefly, a mixture of 150 µL containing 30 µL treated hemolymph, 170 mmol/L CaCl_2_ (30 µL), and 1×PBS (KH_2_PO_4_ 1.76 mmol/L, Na_2_HPO_4_ 10.14 mmol/L, KCl 2.7 mmol/L, NaCl 140 mmol/L; pH 7.0) of 90 µL was preincubated at 30° for 5 min, after which 30 µL of the substrate solution (20 mmol/L L-DOPA) was added, and the mixture was continually incubated at 30° for 10 min. The increase in absorbance at 490 nm was measured using a microplate reader (Thermo Labsystems). The significant variation between control and tested samples was calculated by *t*-test.

### Data availability

The *M. domestica* Illumina reads sequences produced in this study have been submitted to NCBI under project number PRJNA279218, BioSample number SRS885965, and SRA study accession SRP056620, whereas the Transcriptome Shotgun Assembly project has been deposited at DDBJ/EMBL/GenBank under the accession GDAV01000000.

## Results and Discussion

### Illumina RNA-seq and *de novo* assembly

**Figure 1:**
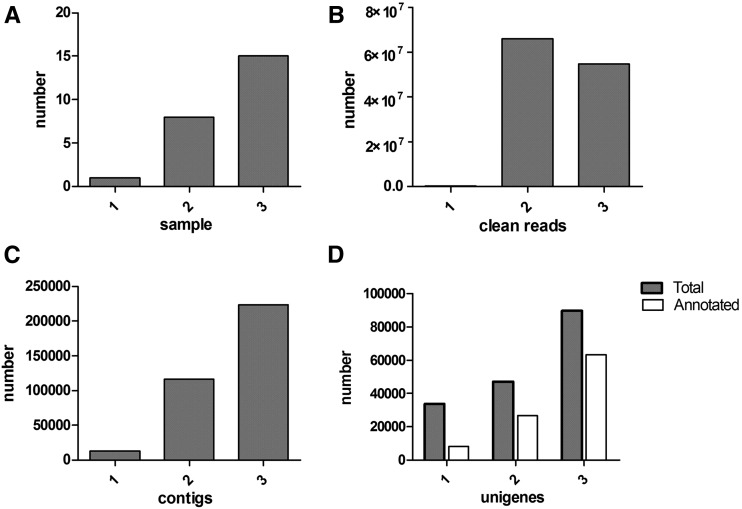
Comparison between different *M. domestica* transcriptomes. (A-D) comparisons of samples, clean reads, contigs, and unigenes of three *M. domestica* transcriptomes marked for 1, 2, and 3 on the abscissa, in which 1 stands for the housefly transcriptome published in 2012 (Liu *et al.* 2012), 2 stands for the transcriptome published in 2014 (Tang *et al.* 2014), and 3 stands for the transcriptome in this study.

To identify the key genes related to the proPO system in *M. domestica*, the combined transcriptome was obtained by Illumina RNA-seq deep sequencing using 15 kinds of samples from housefly larvae, pupae, and adults that were either normal or bacteria-challenged (at 12, 24, and 48 hr) by the mixture of *E. coli* and *S. aureus*. A total of 63,490,654 raw reads were produced from a single run. After filtration, 54,821,138 high-quality clean reads remained and were *de novo* assembled by Trinity into 223,936 contigs. The contigs were further assembled and clustered into 89,842 unigenes with a mean length of 532 nt, which consisted of 30,105 distinct clusters and 59,737 distinct singletons (Table 1). The clean reads and contigs, respectively, were more than 219-fold and nearly 17-fold compared with their counterparts obtained by pyrosequencing housefly larval RNA samples only (Liu *et al.* 2012), whereas contigs and unigenes were all 1.9-fold more than those from another transcriptome constructed by nine kinds of housefly samples, including normal eggs, larvae, pupae, and adults, as well as bacteria-challenged larvae (at 6, 24, 48 hr) (Tang *et al.* 2014). Similarly, the contigs increased to 15-fold in comparison with that of the third transcriptome prepared only by insecticide-resistant adult flies (Li *et al.* 2013). Therefore, this *M. domestica* transcriptome possessed the most comprehensive data compared with previous transcriptomes (Figure 1, Table 2).

**Table 1:** Summary for *Musca domestica* combined transcriptome

Parameters	Output of RNA-seq	Parameters	Assembly Quality
Total no. of raw reads	63,490,654	Total no. of contigs	223,936
Total no. of clean reads	54,821,138	Mean length of contigs (nt)	258
Mean length of clean reads (nt)	90	N50 of contigs (nt)	326
Total clean nucleotides*a* (nt)	4,933,902,420	Total no. of unigenes	89,842
Q20 percentage	96.25%	Mean length of unigenes (nt)	532
N percentage	0.00%	N50 of unigenes (nt)	665
GC percentage	53.69%	Distinct clusters	30,105
		Distinct singletons	59,737

a Total clean nucleotides = total clean reads 1 × reads 1 size + total clean reads 2 × reads 2 size.

**Table 2:** Comparison between different *M. domestica* transcriptomes

Parameters	This Article	Liu *et al.* 2012	Tang *et al.* 2014
Samples	Larvae, pupae, and adults that were either normal or bacterial-challenged; 15	Larvae challenged by bacteria at 12 hr; only 1	Normal eggs, larvae, pupae, adults, and bacterial-challenged larvae at 6, 24, and 48 hr; 9
Clean reads	54,821,138	236,224	66,049,270
Contigs	223,936	13,206	116,687
Unigenes	89,842	33,762	47,086

Of the 89,842 unigenes, 67,349 (75%) had reliable CDS. Of them, 60,877 (67.8%) CDSs were predicted by BLASTx, and 6472 (7.2%) CDSs that had no hit BLAST were further predicted using ESTScan (Table 3).

**Table 3:** Statistical results of CDS numbers

Parameter	No. of CDS	Percentage of CDS
Blastx predicted	60,877	67.8%
ESTscan predicted	6,472	7.2%
Total predicted	67,349	75%

**Figure 2:**
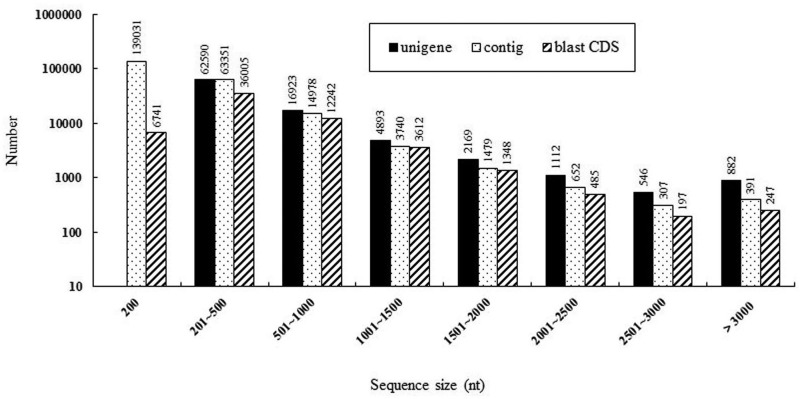
Length distribution of contigs, unigenes, and BLAST CDSs in *M. domestica* transcriptomic library.

In current research, most contigs ranged from 200 nt to 3000 nt, and the lengths of unigenes ranged mostly from 300 nt to 3000 nt after the short unigenes (<300 nt) are removed. The numbers of contigs and unigenes decreased as their sequence sizes increased (Figure 2). To illustrate, the amount of unigenes longer than 500 nt was 36,190 (40.28%), whereas that longer than 1000 nt declined to 12,408 (13.8%). Similarly, 36,005 CDSs ranged from 201 nt to 500 nt, whereas only 247 CDSs were longer than 3 kb. It is worth mentioning that 391 contigs (>3 kb) are approximately 49-times that in published transcriptome (Liu *et al.* 2012).

The assembly quality of the unigenes in this transcriptome database was demonstrated by sequencing nine selected unigenes (Table 4), which showed that the coincidence rates between unigenes and sequencing segments were more than 98%, suggesting that the transcriptomic data were highly reliable.

**Table 4:** Sequencing results of selected unigenes

Unigene ID	Uni-length (nt)	Seq-length (nt)	Identity (nt, %)	Primers	Primer Seqence (5′-3′)
CL4876.Contig1	965	591	99	F	AATCGCAAAGGTCATCATACATC
				R	GACCACGCGTATCGATGTCGAC
CL9802.Contig1	1479	781	99.8	F	TGAGGGACACGGTTCAAGAC
				R	TCCGCACATCATACTGGGG
CL2964.Contig4	2574	109	100	F	TCAGGTGAAGCCTTACAAGTCTC
				R	TGCGAGTCATCATAACCATTG
CL5316.Contig7	1383	259	99	F	TGATAATCTTGCCCAACTCTC
				R	TTGTGAATAACCTTGGAGACC
CL8948.Contig2	1421	240	100	F	AGCCATTGAACAGAAACG
				R	CTTGGCGAGCATTACG
CL572.Contig6	2215	200	100	F	CTACACCTTGCCTCAATTGC
				R	TGGTATAGGTGAAGGGTGTGTG
Unigene14891	1116	298	98	F	AAGATGATAATAAGAAGTGGAGGGC
				R	CGTTGGATTTCTCCTCTTTC
Unigene40871	786	664	98.7	F	AAACCCTGAAACGCCACAAC
				R	TTGCCTTCCAGGGCTTTC
CL9042.Contig2	1194	366	100	F	GGACTTTGAACAGGAAATGGC
				R	CCACCGATCCAGACGGAGTA

### Functional annotation of unigenes

All 89,842 unigene sequences were aligned by BLASTx to the protein databases of Nr, Swiss-Prot, KEGG, COG, and GO and to the nucleotide database of Nt using an E-value cut-off of 10^−5^. A total of 63,237 unigenes (70.4%) were annotated with known genes against Nr (60,353; 67.2%), Nt (32,818; 36.5%), Swiss-Prot (46,084; 51.3%), KEGG (41,578; 46.3%), COG (17,549; 19.5%), and GO databases (44,346; 49.4%) (Table 5). The remaining unigenes (26,605, 29.6%) that could not be annotated to the existing databases might be potential sources of novel genes. Compared to previous reports in which the annotated unigenes, respectively, were 8166 (24.19%) against Nr (Liu *et al.* 2012) and 27,021 (57.39%) in total against Nr, Swiss-Prot, KEGG, and COG (Tang *et al.* 2014), this study provides the most annotated unigenes of 63,237 (70.4%).

**Table 5:** The statistics of annotated unigenes

Parameter	NR	NT	Swiss-Prot	KEGG	COG	GO	All
Annotated unigenes	60,353	32,818	46,084	41,578	17,549	44,346	63,237
Percentage	67.2%	36.5%	51.3%	46.3%	19.5%	49.4%	70.4%

**Figure 3:**
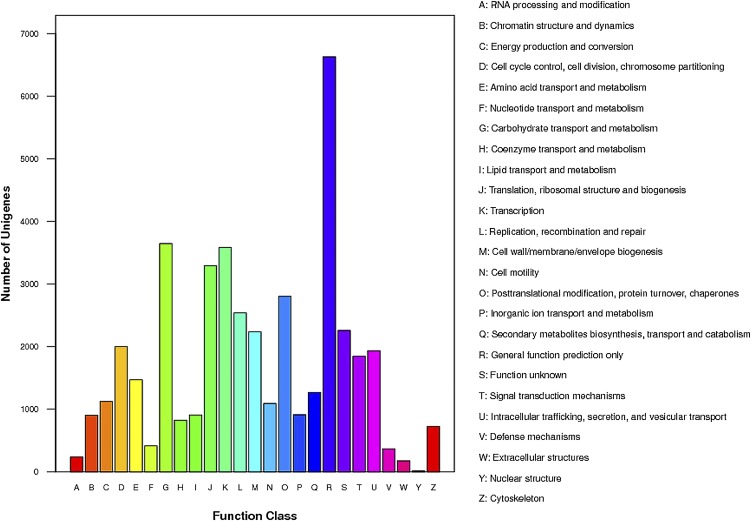
COG annotations of putative proteins. All putative proteins were classified functionally into 25 categories marked as A-W and Y-Z based on their COG annotations. Of them, the R group was the largest category and accounted for 37.76%, followed by groups G (20.76%) and K (20.42%). In contrast, those marked as Y (0.097%), W (0.99%), and A (1.35%) belonged to the smallest groups.

As shown in Figure 3, the 17,549 unigenes were classified into 25 COG functional categories, which was the same as two previous reports of *D. antiqua* (Zhang *et al.* 2014) and *M. domestica* (Tang *et al.* 2014). Among 25 COG categories, the largest one was general function prediction with the biggest gene numbers and percentage (6627; 37.76%), followed by carbohydrate transport and metabolism (3644; 20.76%) and transcription (3584; 20.42%). The smallest cluster was nuclear structure (17; 0.097%). The cluster of defense mechanisms (366; 2.09%) listed fourth from the bottom, which had considerable genes in comparison with that of *D. antiqua* (109; 1.7%) (Zhang *et al.* 2014) and *M. domestica* (279; 1.2%) (Tang *et al.* 2014).

**Figure 4:**
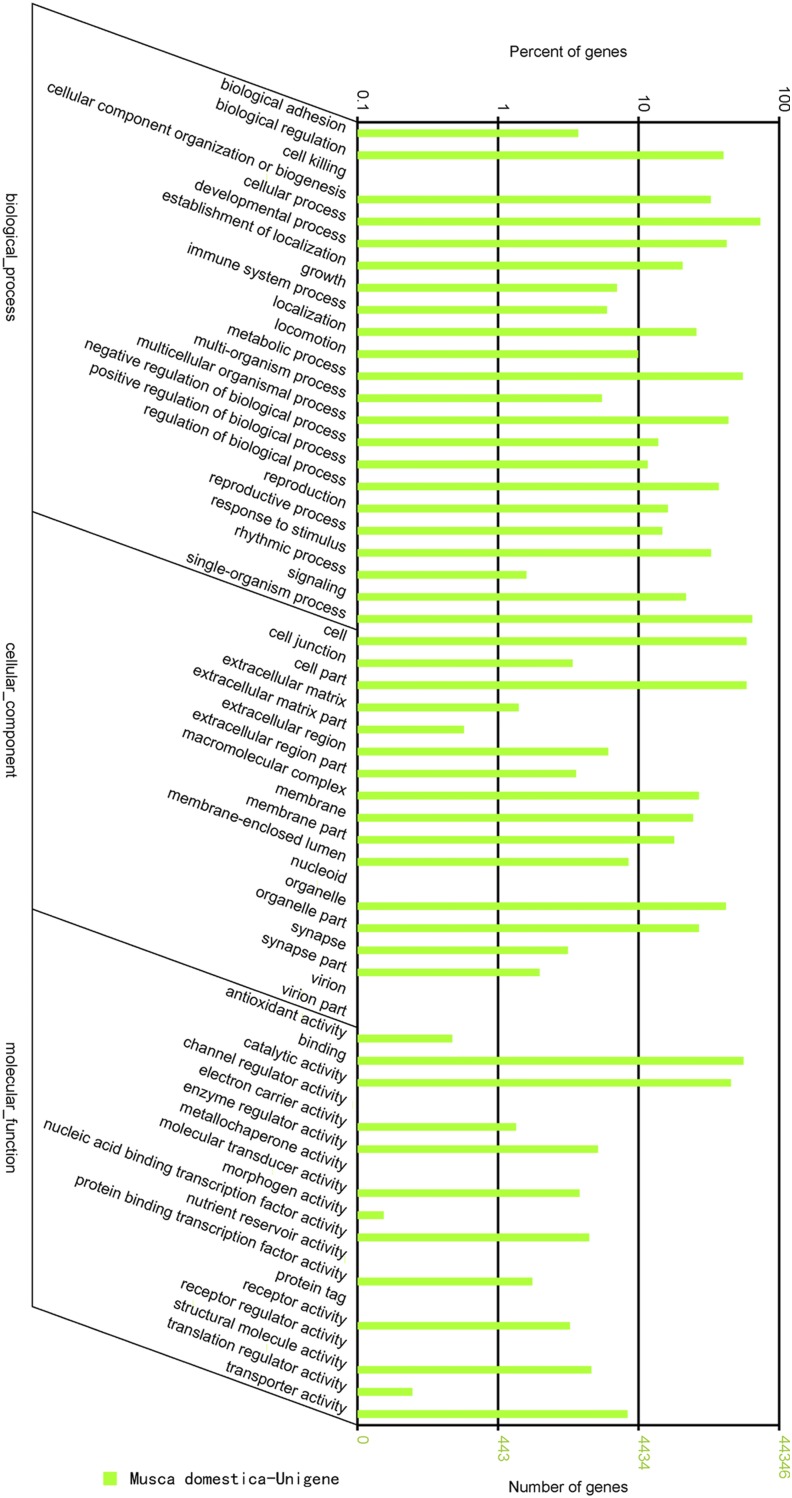
GO classification of predicted genes. Three GO categories of genes including biological process, cellular component, and molecular function were presented. The numbers of genes in a given functional group are shown to the right of the *y*-axis and the percentages are marked to the left of the *y*-axis.

Of the 60,353 Nr hits, GO terms were assigned to 44,346 unigenes (49.4% of total) for functional categorization, which were divided into 59 functional groups, and were allocated into three main categories, including biological process, cellular component, and molecular function (Figure 4). The top five belonged to the groups of cellular process (32,548; 73.40%), single-organism process (28,495; 64.26%), cell and cell part (26,014; 58.66%), binding (24,663; 55.61%), and metabolic process (24,372; 54.96%). Seven groups including nucleoid, virion, virion part, channel regulator activity, metallochaperone activity, morphogen activity, and nutrient reservoir activity had more than 10 but less than 100 genes. No more than 10 genes were clustered to the groups of protein tag, cell killing, and receptor regulator activity.

By searching the KEGG database with the 89,842 unigenes, a total of 41,578 unigenes (46.3%) were mapped to 258 KEGG pathways (Table S2). The most representative pathways were metabolic pathways (5704 unigenes; 13.72%), followed by the cancer (5704; 3.8%), focal adhesion (1469; 3.5%), regulation of actin cytoskeleton (1289; 3.1%), and RNA transport pathways (1221; 2.9%). Notably, a subset of unigenes was assigned to some immune-related pathways, including coagulation cascades and signaling transduction pathways.

In comparison to previous housefly transcriptome published in 2014 (Tang *et al.* 2014) in which only 53.39% (27,021) unigenes were annotated and less functional annotation information were provided, including 25 COG categories, 48 GO groups, and 239 KEGG pathways, this transcriptome possesses a more abundant transcript information.

### Identification of key genes related to the proPO system

On the basis of functional annotations of 89,842 unigenes and similarity search to known genes, a batch of unigenes that might be full lengths or parts of several putative key genes involved in the *M. domestica* proPO system was harvested. After artificial sequence splicing through the removal of very short unigenes, *i.e.*, those without important structural domains or significant hits, a total of 66 unigenes remained and underwent further sequence analyses and phylogenetic analyses. As a result, these unigenes were clustered into different groups with known genes of *PGRPs*, *proPOs*, *PAPs*, and *Serpins*, suggesting *M. domestica* might have these analogs, called as *mdPGRPs*, *mdproPOs*, *mdPAPs*, and *mdSerpins* (Table 6).

**Table 6:** Putative genes identified in connection with proPO system in *M. domestica* transcriptome

Unigene ID	Nucleotide Size (nt)	Predicted Protein Domains*^a^*	Signal Peptide*^b^*	Hit-ACC [Species]	E-value	Annotated Function	Putative Gene*^c^*
		***Peptidoglycan recognition receptor of M. domestica (MdPGRP)***					
Unigene46163	687	PGRP(27-169); Aim_2(40-175)	1-23	Q9VYX7 [*Drosophila melanogaster*]	8e-64	PGRP SA	*MdPGRP SA*
Unigene46164	685	PGRP(35-177); Aim_2(48-183)	1-24	Q9VYX7 [*Drosophila melanogaster*]	2e-68	PGRP SA	*MdPGRP SA*
Unigene653	682	PGRP(35-177); Aim_2(48-183)	1-24	Q9VYX7 [*Drosophila melanogaster*]	2e-69	PGRP SA	*MdPGRP SA*
CL5483.Contig1	719	PGRP(22-165); Aim_2(33-171)	1-21	Q70PY2 [*Drosophila melanogaster*]	8e-78	PGRP SB1	*MdPGRP SB*
CL5483.Contig2	672	PGRP(21-164); Aim_2(32-170)	1-20	Q70PY2 [*Drosophila melanogaster*]	1e-79	PGRP SB1	*MdPGRP SB*
CL5483.Contig3	696	PGRP(21-164); Aim_2(32-170)	1-20	Q70PY2 [*Drosophila melanogaster*]	4e-79	PGRP SB1	*MdPGRP SB*
Unigene41919	646	PGRP(20-162); Aim_2(33-168)	1-19	ADI87391.1 [*Lucilia sericata*]	2e-71	PGRP	*MdPGRP SC*
CL9661.Contig2	593	PGRP(22-164); Aim_2(31-170)	1-21	ADI87391.1 [*Lucilia sericata*]	1e-78	PGRP	*MdPGRP SC*
CL4993.Contig1	593	PGRP(22-164); Aim_2(33-170)	1-21	ADI87391.1 [*Lucilia sericata*]	1e-89	PGRP	*MdPGRP SC*
CL4993.Contig2	664	PGRP(22-164); Aim_2(33-170)	1-21	ADI87391.1 [*Lucilia sericata*]	1e-94	PGRP	*MdPGRP SC*
Unigene25719	829	PGRP(27-170); Aim_2(44-176)	1-25	XP_002094076.1 [*Drosophila yakuba*]	4e-46	PGRP SD	*MdPGRP SD*
Unigene34801	718	PGRP(24-167); Aim_2(39-173)	1-22	Q70PR8.1 [*Drosophila simulans*]	5e-45	PGRP SD	*MdPGRP SD*
CL175.Contig1	536	PGRP(24-157); Aim_2(42-157)	1-22	Q70PR8.1 [*Drosophila simulans*]	9e-30	PGRP SD	*MdPGRP SD*
CL3331.Contig2	776	PGRP(102-244); Aim_2(116-250)	—	XP_002084233.1 [*Drosophila simulans*]	1e-89	PGRP LA	*MdPGRP LA*
CL5285.Contig1	801	PGRP(22-165); Aim_2(33-171)	1-17	DQ307160.1 [*Glossina morsitans morsitans*]	2e-14	PGRP LB	*MdPGRP LB*
CL5285.Contig2	784	PGRP(23-166); Aim_2(34-172)	—	DQ307160.1 [*Glossina morsitans morsitans*]	2e-14	PGRP LB	*MdPGRP LB*
Unigene47733	1076	PGRP(166-309); Aim_2(180-315)	—	DQ307161.1 [*Glossina morsitans morsitans*]	2e-08	PGRP LC	*MdPGRP LC*
CL7750.Contig1	834	PGRP(177-254)	—	NP_648298.1 [*Drosophila melanogaster*]	2e-49	PGRP LCb	*MdPGRP LC*
CL7750.Contig2	771	PGRP(156-233)	—	ABC25065.1 [*Glossina morsitans morsitans*]	1e-41	PGRP LC	*MdPGRP LC*
Unigene46613	1366	PGRP(166-309); Aim_2(178-315)	—	Q9VXN9 [*Drosophila melanogaster*]	6e-69	PGRP LE	*MdPGRP LE*
Unigene35645	601*^d^*	PGRP(1-133), Aim_2(2-139)	—	Q9VXN9 [*Drosophila melanogaster*]	1e-44	PGRP LE	*MdPGRP LE*
		***Prophenoloxidase of M. domestica (MdproPO)***					
CL572.Contig2	759	Hemocyanin_M(2-140); Tyrosinase(8-144); Hemocyanin_C(146-229)	—	AAR84669.1 [*Musca domestica*]	5e-123	proPO	*MdproPO 1*
CL572.Contig3	1291	Hemocyanin_M(3-140); Tyrosinase(9-144); Hemocyanin_C(146-402)	—	AAR84669.1 [*Musca domestica*]	0.0	proPO	*MdproPO 1*
CL572.Contig4	2255	Hemocyanin_N(2-122); Hemocyanin_M(101-395); Tyrosinase(177-399); Hemocyanin_C(401-657)	—	AF161260.1 [*Neobellieria bullata*]	0.0	proPO 1	*MdproPO 1*
CL572.Contig5	1628	Hemocyanin_M(1-213); Tyrosinase(4-217); Hemocyanin_C(219-475)	—	AF161260.1 [*Neobellieria bullata*]	0.0	proPO 1	*MdproPO 1*
CL572.Contig6	2215	Hemocyanin_N(4-142); Hemocyanin_M(120-415); Tyrosinase(197-419); Hemocyanin_C(421-677)	—	AAR84669.1 [*Musca domestica*]	0.0	proPO	*MdproPO 1*
Unigene49966	1668	Hemocyanin_M(1-213); Tyrosinase(4-217); Hemocyanin_C(219-475)	—	AF161260.1 [*Neobellieria bullata*]	0.0	proPO 1	*MdproPO 1*
Unigene33273	2190	Hemocyanin_N(24-145); Hemocyanin_M(149-416); Tyrosinase(197-420); Hemocyanin_C(422-682)	—	AF161261.1 [*Neobellieria bullata*]	0.0	proPO 2	*MdproPO 2*
Unigene9393	1330	Hemocyanin_M(2-191); Tyrosinase(53-195); Hemocyanin_C(197-420)	—	AF161261.1 [*Neobellieria bullata*]	0.0	proPO 2	*MdproPO 2*
		***Prophenoloxidase activating factor of M. domestica (MdPAP)***					
Unigene46765	1297	CLIP(42-95);Tryp_SPc(132-391)	1-25	P13582 [*Drosophila melanogaster*]	3e-148	Serine protease easter	*MdPAP 1*
CL4801.Contig2	1334	CLIP(30-83);Tryp_SPc(108-367)	1-24	P13582 [*Drosophila melanogaster*]	4e-104	Serine protease easter	*MdPAP 1*
CL1201.Contig1	1491	CLIP(51-104);Tryp_SPc(142-381)	1-34	P13582 [*Drosophila melanogaster*]	1e-150	Serine protease easter	*MdPAP 1*
CL1201.Contig2	1190	CLIP(42-95);Tryp_SPc(132-364)	1-25	P13582 [*Drosophila melanogaster*]	2e-152	Serine protease easter	*MdPAP 1*
CL2563.Contig1	1271	CLIP(29-88);Tryp_SPc(120-380)	1-21	P13582 [*Drosophila melanogaster*]	5e-105	Serine protease easter	*MdPAP 1*
CL9802.Contig1	1479	CLIP(39-91);Tryp_SPc(118-368)	1-35	P13582 [*Drosophila melanogaster*]	7e-70	Serine protease easter	*MdPAP 2*
CL9736.Contig2	1255	CLIP(39-93);Tryp_SPc(142-391)	1-35	P13582 [*Drosophila melanogaster*]	2e-69	Serine protease easter	*MdPAP 2*
CL4876.Contig1	965	Tryp_SPc(52-278)	1-27	ACC93936.1 [*Musca domestica*]	3e-94	Prophenoloxidase activating factor	*MdPAP 3*
CL4876.Contig2	974	Tryp_SPc(53-279)	1-29	ACC93936.1 [*Musca domestica*]	1e-93	Prophenoloxidase activating factor	*MdPAP 3*
CL4876.Contig3	1006	Tryp_SPc(53-283)	1-29	ACC93936.1 [*Musca domestica*]	2e-68	Prophenoloxidase activating factor	*MdPAP 3*
CL14909.Contig1	1730	CLIP(36-92,115-168);1Tryp_SPc(204-447)	1-23	P21902 [*Tachypleus tridentatus*]	2e-59	Proclotting enzyme	*MdPAP 3*
CL16739.Contig2	1584	CLIP(3-59,141-194);Tryp_SPc(232-476)	—	P21902 [*Tachypleus tridentatus*]	9e-58	Proclotting enzyme	*MdPAP 3*
		***Serine protease inhibitors of M. domestica (MdSerpin)***					
Unigene46543	1509	Serpin(40-403)	1-24	ABC25072.1 [*Glossina morsitans morsitans*]	3e-124	Serine protease inhibitor 1	*MdSerpin 1*
CL1146.Contig4	1298	Serpin(38-402)	1-17	ABC25072.1 [*Glossina morsitans morsitans*]	2e-123	Serine protease inhibitor 1	*MdSerpin 1*
Unigene48882	1277	Serpin(41-395)	1-27	XP_005177806.1 [*Musca domestica*]	0.0	Antitrypsin-like	*MdSerpin 2*
CL5316.Contig7	1383	Serpin(53-410)	1-28	XP_005177808.1 [*Musca domestica*]	4e-162	Serine protease inhibitor 3/4-like	*MdSerpin 3*
CL8948.Contig2	1421	Serpin(65-420)	—	XP_005177808.1 [*Musca domestica*]	0.0	Serine protease inhibitor 3/4-like	*MdSerpin 3*
CL8948.Contig3	1508	Serpin(65-419)	—	XP_005177808.1 [*Musca domestica*]	0.0	Serine protease inhibitor 3/4-like	*MdSerpin 3*
Unigene47907	1122	Serpin(1-334)	—	XP_005177808.1 [*Musca domestica*]	2e-147	Serine protease inhibitor 3/4-like	*MdSerpin 3*
Unigene3626	923	Serpin(38-291)	1-24	XP_005177808.1 [*Musca domestica*]	2e-88	Serine protease inhibitor 3/4-like	*MdSerpin 4*
CL9667.Contig1	1565	Serpin(52-422)	1-20	ABC25075.1 [*Glossina morsitans morsitans*]	0.0	Serine protease inhibitor 5	*MdSerpin 5*
Unigene2774	1548	Serpin(87-450)	1-23	XP_005183089.1 [*Musca domestica*]	0.0	Serpin B3-like	*MdSerpin 6*
Unigene40627	1248	Serpin(32-378)	1-20	XP_005179838.1 [*Musca domestica*]	0.0	Serine protease inhibitor 3/4-like isoform X2	*MdSerpin 6*
CL11280.Contig1	759	Serpin(1-149)	—	XP_005187409.1 [*Musca domestica*]	2e-64	Leukocyte elastase inhibitor-like	*MdSerpin 6*
CL10810.Contig2	1189	Serpin(24-163)	1-20	XP_004533758.1 [*Ceratitis capitata*]	3e-72	Serpin B4-like	*MdSerpin 7*
Unigene49496	1670	Serpin(66-437)	1-23	XP_005183496.1 [*Musca domestica*]	0.0	Plasminogen activator inhibitor 2-like	*MdSerpin 8*
CL13613.Contig2	774	Serpin(1-182)	—	XP_005183496.1 [*Musca domestica*]	2e-66	Plasminogen activator inhibitor 2-like	*MdSerpin 8*
Unigene42869	732	Serpin(1-184)	—	ABC25079.1 [Glossina morsitans morsitans]	6e-61	Serine protease inhibitor	*MdSerpin 8*
Unigene45821	777	Serpin(17-237)	—	XP_005185733.1 [*Musca domestica*]	2e-170	Leukocyte elastase inhibitor-like	*MdSerpin 9*
CL1905.Contig2	1942	Serpin(75-622)	1-27	XP_005188485.1 [*Musca domestica*]	4e-121	Leukocyte elastase inhibitor B-like	*MdSerpin 9*
CL530.Contig1	1604	Serpin(49-418)	1-19	XP_005191108.1 [*Musca domestica*]	0.0	Serpin B6-like	*MdSerpin 10*
CL7972.Contig1	802	Serpin(1-215)	—	XP_005182902.1 [*Musca domestica*]	4e-107	Uncharacterized protein LOC101899860	*MdSerpin 11*
Unigene32587	735	Serpin(1-217)	—	XP_005182902.1 [*Musca domestica*]	4e-163	Uncharacterized protein LOC101899860	*MdSerpin 11*
Unigene34684	750	Serpin(1-233)	—	XP_005188485.1 [*Musca domestica*]	7e-95	Leukocyte elastase inhibitor B-like	*MdSerpin 11*
Unigene1407	1017	Serpin(1-302)	—	XP_005183089.1 [*Musca domestica*]	0.0	serpin B3-like	*MdSerpin 11*
Unigene14891	1116	Serpin(85-351)	1-29	XP_005183089.1 [*Musca domestica*]	0.0	Serpin B3-like	*MdSerpin 11*
Unigene9084	476	Serpin(1-145)	—	XP_005185733.1 [*Musca domestica*]	4e-57	Leukocyte elastase inhibitor-like	*MdSerpin 11*

a The protein domains and *^b^*signal peptide are predicted by available nucleotide sequences, respectively.

c The putative genes were provided mainly with reference to unigene annotation and unigene phylogenetic analysis (see Figure 5, Figure 6, Figure 7, Figure 8, and Figure S1).

d The number indicates the length of splicing nucleotide sequences of Unigene35644 and Unigene35645.

#### *MdPGRP* genes:

Previous studies have demonstrated that PGRPs can sense and bind peptidoglycan (PGN) of invading microbial pathogens and subsequently initiate the proPO cascade and melanization in insects (Kurata 2014; Yoshida *et al.* 1996). So, it is very important to identify *mdPGRP* genes for exploring the activating mechanism of the proPO system in *M. domestica*.

Since PGRPs were first purified from the silkworm (*B. mori*) hemolymph (Yoshida *et al.* 1996), up to 19 PGRPs have been identified and categorized into two groups of short type (PGRP-S) and long type (PGRP-L) in insects. The short PGRPs have a signal peptide, whereas long PGRPs may lack an export signal but have some transmembrane domains. In addition, PGRP genes are conservative, with at least one PGRP domain similar to N-acetylmuramyl-alanine amidases from insects to mammals (Kurata 2014).

As expected, 21 putative *mdPGRP* unigenes all have one PGRP domain by SMART analysis in the present study. With the exception of the PGRP domain, the vast majority of unigenes also contain a predicted overlapped amidase domain at their C-terminus. Phylogenetic analyses showed that 21 mdPGRPs were clustered into eight groups with 14 DmPGRPs of *D. melanogaster*, in which each group contains at least one specific *DmPGRP* gene, suggesting *M. domestica* may possess eight different *mdPGRP* genes (Figure 5). Interestingly, the eight putative *mdPGRP* genes were also sorted into short type (*mdPGRP-SA*, -*SB*, -*SC*, and -*SD*) and long type (*mdPGRP-LA*, -*LB*, -*LC*, and -*LE*), as detected in *D. melanogaster* (Kurata 2014). Compared with previous studies including transcriptomes (Liu *et al.* 2012; Tang *et al.* 2014) and whole genome (Scott *et al.* 2014), in which the *PGRP* genes separately were one, 16, and nine in *M. domestica*, it is acceptable for *M. domestica* to have eight putative *mdPGRP* genes in this study.

**Figure 5:**
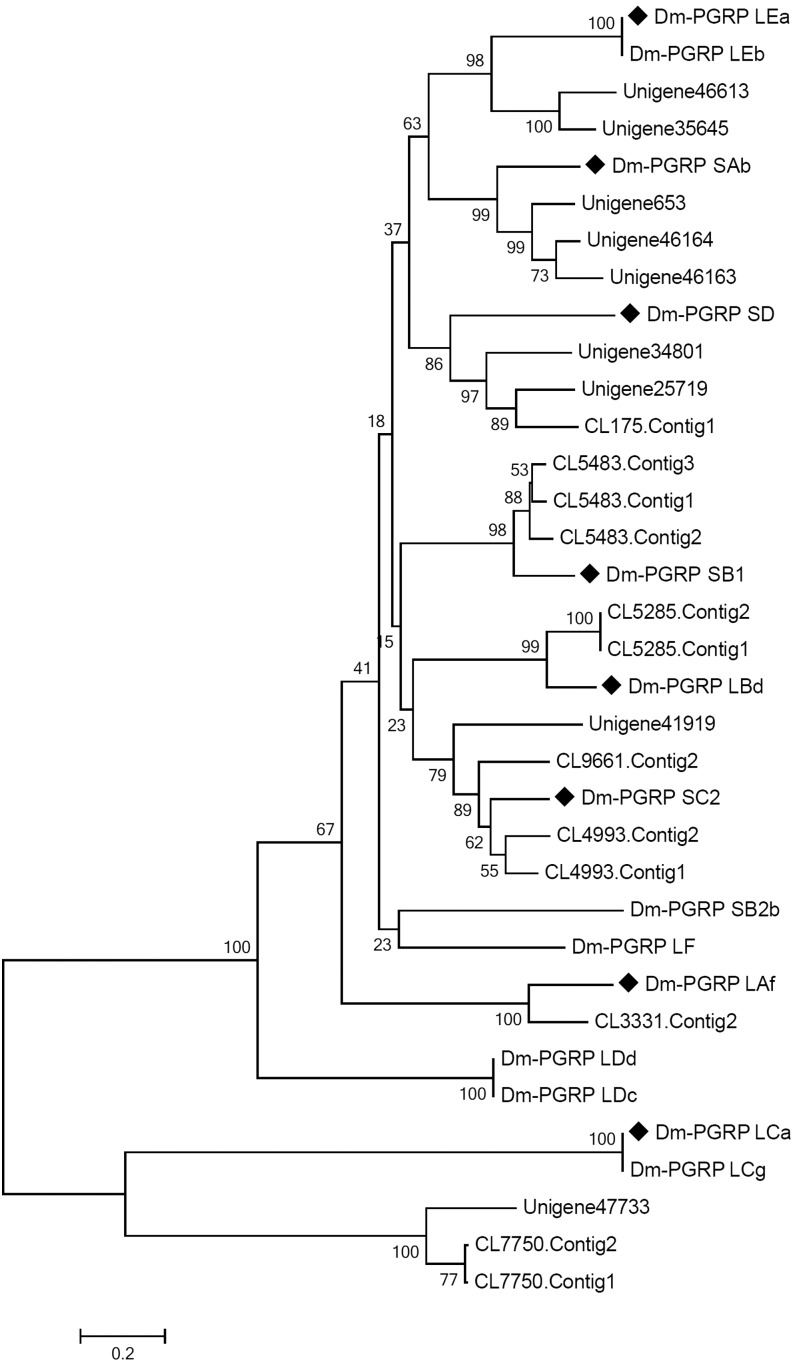
Phylogenetic analysis of mdPGRP unigenes. The amino acid sequences from 21 mdPGRP unigenes of *M. domestica* and 14 PGRP genes of *D. melanogaster* including Dm-PGRP SAb (AHN59598), Dm-PGRP SB1 (AAF49420), Dm-PGRP SB2b (AGB94663), Dm-PGRP SC2 (AAF59051), Dm-PGRP SD (AAF50530), Dm-PGRP LAf (AAF50303), Dm-PGRP LBd (AFH06370), Dm-PGRP LCa (AAF50302), Dm-PGRP LCg (ACZ94669), Dm-PGRP LDc (AAO41277), Dm-PGRP LDd (ACL83248), Dm-PGRP LEa (AAF48519), Dm-PGRP LEb (AFH07408), and Dm-PGRP LF(AAF50301) were used to build the NJ phylogenetic tree by MEGA 5.0 with 1000 bootstraps. The 21 mdPGRP unigenes were clustered into eight groups, with each group containing at least one specific Dm-PGRP gene (marked diamond block).

It is notable that the *mdPGRP-LE* gene may not have amidase activity, because the sequence alignment indicated that both mdPGRP-LE and DmPGRP lacked the equally critical catalytic residues in their amidase domain (data not shown), suggesting mdPGRP-LE might be the initiator of the proPO activation cascade, as determined in other insects (Kurata 2014; Tindwa *et al.* 2013).

#### *MdproPO* genes:

**Figure 6:**
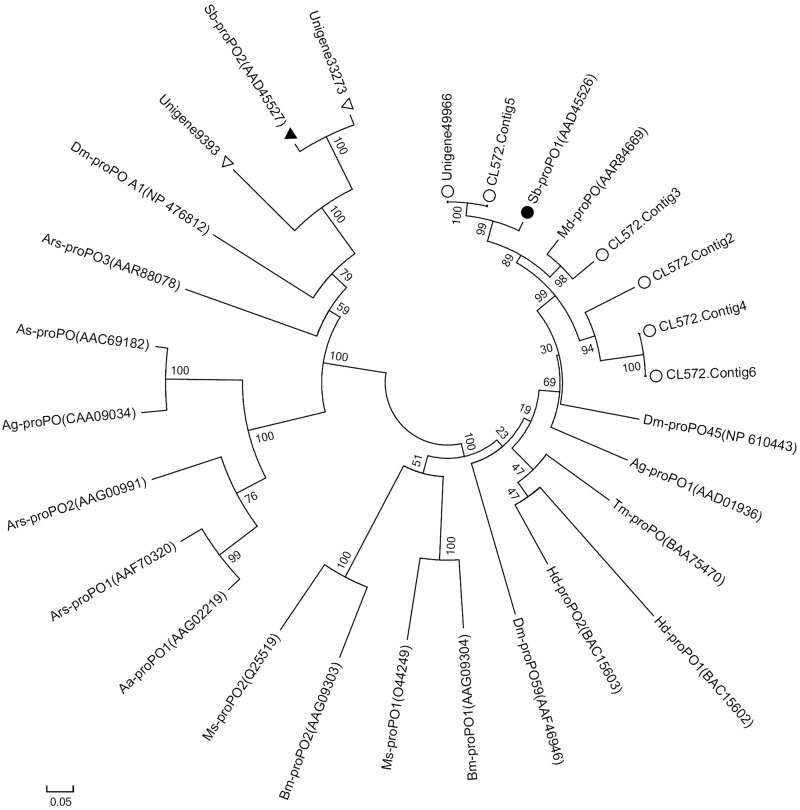
Phylogenetic analysis of mdproPO unigenes. The amino acid sequences from eight unigenes of mdproPO in *M. domestica* and 21 proPO genes in other species including *M. domestica* (Md, 1), *Sarcophaga bullata* (Sb, 2), *Drosophila melanogaster* (Dm, 3), *Anopheles gambiae* (Ag, 2), *Anopheles stephensi* (As,1), *Armigeres subalbatus* (Ars, 3), *Aedes aegypti* (Aa, 1), *Manduca sexta* (Ms, 2), *Bombyx mori* (Bm, 2), *Tenebrio molitor* (Tm, 1), *Holotrichia diomphalia* (Hd, 2), and *Eriocheir sinensis* (Es, 1) were used to build the NJ phylogenetic tree by MEGA 5.0 with 1000 bootstraps. Six unigenes (circle) were clustered with Sb-proPO1 (disc) into group 1, and the other two unigenes (triangles) were clustered with Sb-proPO2 (triangle block) into group 2.

Since the *proPO* sequences of *M. sexta* and *B. mori* were reported in the 1990s, the number of *proPO* genes has been more than 20 in insects (Zhu *et al.* 2011). Most insect species contain two *proPO* genes, as in *M. sexta* and *B. mori*, but in *D. melanogaster* the number of *proPO* genes gets three (Ross *et al.* 2003) and in *A. gambiae* it becomes nine (Christophides *et al.* 2002). According to whether the encoded proteins contain the representative domains like Hemocyanin M, Tyrosinase, Hemocyanin C, and Hemocyanin N, eight *proPO* unigenes were picked out from this housefly transcriptomic database and clustered into two groups with 21 known *proPO* genes of other insect species through phylogenetic analyses. Of them, six selected unigenes and the *SbproPO1* gene of *Sarcophaga bullata* were assigned to the same group, and the other two unigenes and the *SbproPO2* gene of *S. bullata* were assigned to another group (Figure 6), suggesting that *M. domestica* at least has two sequential diverged *mdproPO* genes, defined as *mdproPO1* and *mdproPO2*. In recent years, *M. domestica* had been reported to have seven or eight *proPO* transcripts, respectively, in transcriptome (Tang *et al.* 2014) and whole genome (Scott *et al.* 2014). Therefore, this study is consistent with previous reports.

#### *MdPAP* genes:

**Figure 7:**
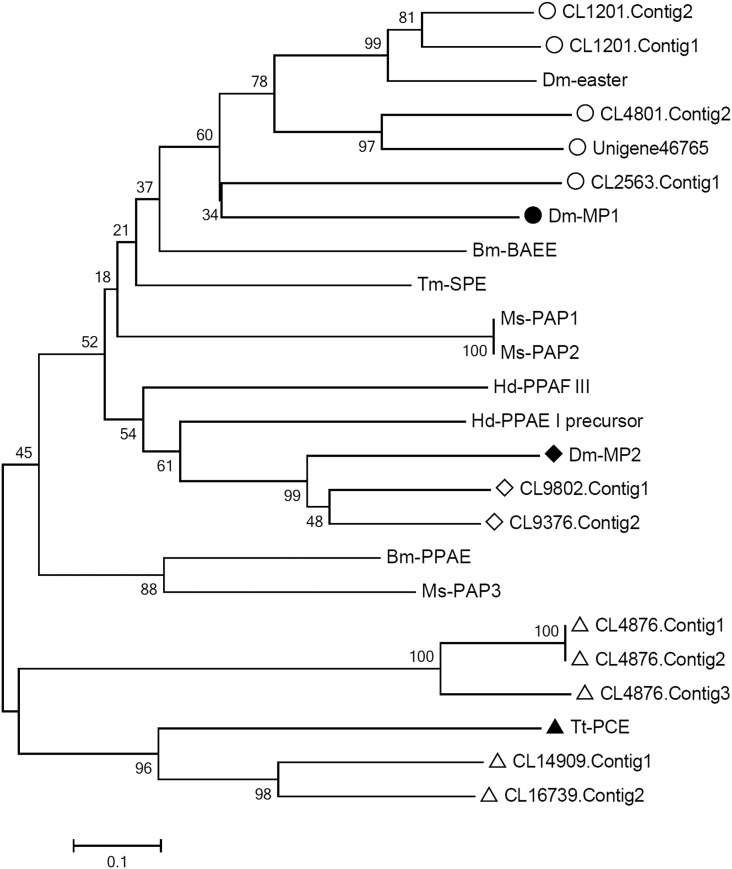
Phylogenetic analysis of mdPAP unigenes. The amino acid sequences from 12 mdPAP unigenes in *M. domestica* and 12 known genes of prophenoloxidase activating factor in other species including *Drosophila melanogaster* (Dm-easter AAF55170; Dm-MP1 AAF52151.3; Dm-MP2 AAF54143.1), *Holotrichia diomphalia* (Hd-PPAE I precursor BAA34642.1; Hd-PPAF III BAC15604.1), *Tenebrio molitor* (Tm-SPE BAG14262.2), *Bombyx mori* (Bm-BAEE NP_001036844.1; Bm-PPAE NP_001036832.1), *Manduca sexta* (Ms-PAP1 AAX18636.1; Ms-PAP2 AAL76085.1; Ms-PAP3 AAO74570.1), and *Tachypleus tridentatus* (Tt-PCE AAA30094.1) were used to build the NJ phylogenetic tree by MEGA 5.0 with 1000 bootstraps. The circle-marked five unigenes and Dm-easter and Dm-MP1 were clustered into group 1. Two unigenes and one Dm-MP2 (diamond) were divided to group 2. The other five unigenes and Tt-PCE (triangle) were clustered into group 3.

In insects, PAPs are the final key activators in the proPO system and can directly activate proPO into PO by cleaving proPO at an Arg-Phe bond at approximately residue 50 (An *et al.* 2013; Pang *et al.* 2014). At present, several PAPs have been identified from *B. mori* (Satoh *et al.* 1999), *M. sexta* (Jiang *et al.* 2003), *D. melanogaster* (An *et al.* 2013), *Anopheles gambiae* (An *et al.* 2011a), *H. diomphalia* (Kim *et al.* 2002), and *T. molitor* (Kan *et al.* 2008), which typically contain a C-terminal catalytic domain (Tryp_SPc) and one or two N-terminal clip domains, therefore called as clip-domain serine proteases (Jiang and Kanost 2000). In this *M. domestica* transcriptomic database, 14 unigenes were annotated as clip-domain serine proteases, which is more than double that of published transcriptome with six clip-domain serine protease genes (Tang *et al.* 2014). After removing two sequences with uncompleted domains, the remaining 12 unigenes were clustered into three groups together with 12 known PAP genes by phylogenetic analyses (Figure 7), suggesting *M. domestica* might have three PAP genes named as *mdPAP1*, *mdPAP2*, and *mdPAP3*, as described in both *M. sexta* and *H. diomphalia* (Jiang *et al.* 2003; Kim *et al.* 2002). It is notable that no *SPH* gene was retrieved from any transcriptome (Liu *et al.* 2012; Tang *et al.* 2014) as well as genome of *M. domestica* (Scott *et al.* 2014). Thus, we speculated that SPH might be unnecessary for mdPAPs to effectively activate the proPO system in *M. domestica*.

#### *mdSerpin* genes:

**Figure 8:**
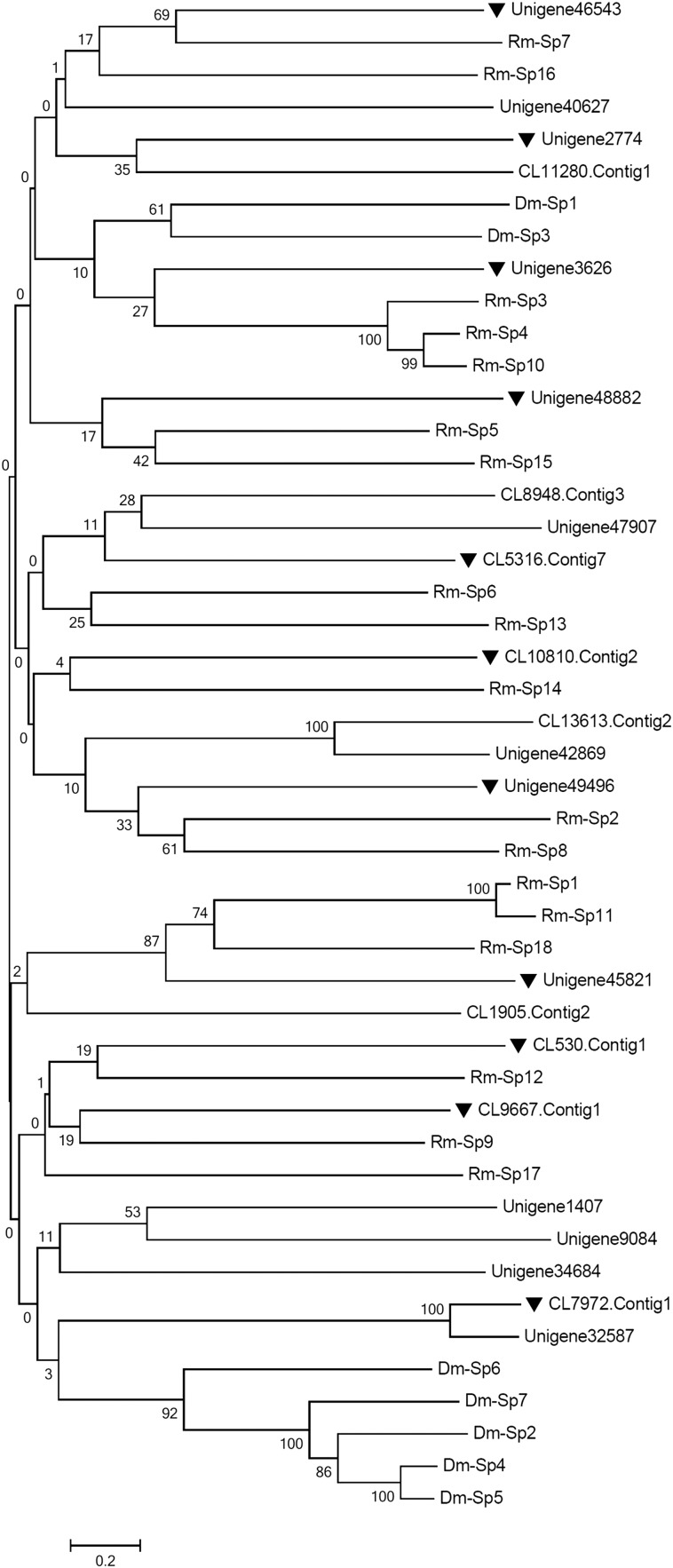
The interspecific phylogenetic analyses of mdSerpin unigenes. The amino acid sequences from 22 mdSerpin unigenes in *M. domestica* and seven Dm-Sps (Dm-Sp1 to Dm-Sp7: FBpp008812, FBpp0079094, FBpp0079171, FBpp0079243, FBpp0080979, FBpp0289586, FBpp0110138) of *Drosophila melanogaster* and 18 Rm-Sps (Rm-Sp1 to Rm-Sp18: AHC98652.1, AHC98653.1, AHC98654.1, AHC98655.1, AHC98656.1, AHC98657.1, AHC98658.1, AHC98659.1, AHC98660.1, AHC98661.1, AHC98662.1, AHC98663.1, AHC98664.1, AHC98665.1, AHC98666.1, AHC98667.1, AHC98668.1, AHC98669.1) of *Rhipicephalus microplus* were used to build the NJ phylogenetic tree by MEGA 5.0 with 1000 bootstraps. Eleven representative unigenes (triangle) were clustered into 11 groups together with known genes of *D. melanogaster* and *R. microplus*.

Serpins have been identified in nearly all species that contain a conserved serpin domain that are usually 350 to 400 amino acid residues long. Some serpins do not have an N-terminal signal sequence and function intracellularly; in contrast, the others have an N-terminal signal sequence and function extracellularly (Gulley *et al.* 2013). In this study, 123 unigenes were annotated as serine proteinase inhibitors or serpins. After being checked manually, 25 unigenes were retrieved with relatively complete serpin domains. To date, more than 10,000 serpin sequences have been published; the *D. melanogaster* genome contains 29 *Dmserpin* genes (Reichhart 2005), whereas the cattle tick *R*. (*Boophilus*) *microplus* has 18 *Rmserpin* genes (Tirloni *et al.* 2014). Combined with the function annotations and the phylogeny analyses of intraspecies (Figure S1) and interspecies (Figure 8), the 25 retained serpin unigenes were divided into 11 categories preliminarily, suggesting *M. domestica* might have at least 11 putative *mdSerpin* genes. Compared with two previous reports in which the number of *Serpin* genes, respectively, was 10 (Tang *et al.* 2014) and 11 (Scott *et al.* 2014), the 11 putative *mdSerpin* genes are acceptable in *M. domestica*.

**Figure 9:**
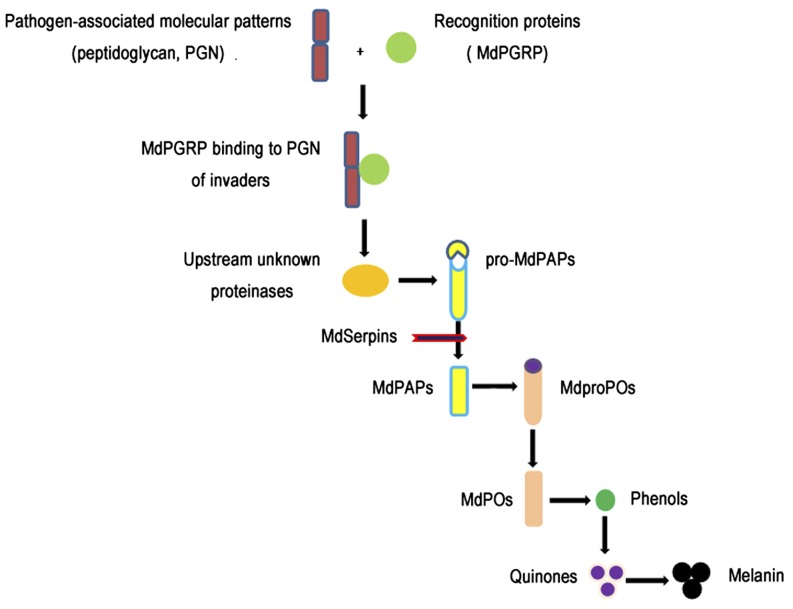
Simplified schemes of the activation of proPO system in *Musca domestica*. The proPO system might be triggered by pattern-recognition proteins such as PGRP that have bound to pathogen-associated molecular patterns like PGN. Then, a proteolytic cascade is activated in which the upstream proteinases are activated by the complex of PGRP and PGN, culminating in the activation of pro-forms of prophenoloxidase-activating enzymes (promdPAPs), which are cleaved into active mdPAPs; some mdPAPs are capable of directly cleaving mdproPOs into active mdPOs and the mdPOs catalyze phenols into quinones that are converted into melanin spontaneously. The activating process may be suppressed by several serine proteinase inhibitors (mdSerpins) that specifically block different steps of the activation cascade.

Altogether, we have identified 24 putative genes through *M. domestica* transcriptomic analyses, including eight *mdPGRP* (*-SA*, *-SB*, *-SC*, *-SD*, *-LA*, *-LB*, *-LC*, *-LE*), two *mdproPO* (*1*–*2*), three *mdPAP* (*1*–*3*), and 11 *mdSerpin* (*1*–*11*), but short of *mdSPH*. We selected nine unigenes that were respectively defined as *mdPGRP* (*LE*, *SC*), *mdproPO1*, *mdPAP* (*1*, *2*, *3*), and *mdSerpin* (*3*, *11*) and conducted an alignment to their matched sequences in the RefSeq database of the *M. domestica* genome (Scott *et al.* 2014). The results indicated that transcript coverage varied from 75% to 100%, the average was 91% of their length, and the e-values of 98% alignments were 0.0 (Table 7). Therefore, it is reasonable for us to infer that *M. domestica* might have such a novel proPO system composed of these candidate genes, as illustrated in Figure 9.

**Table 7:** Sequence alignments between unigenes used in qRT-PCR and matched sequences in RefSeq RNA database

Putative Gene	Unigene ID	Hit ID	Query Cover	E-value	Identity	Genome Function
*MdPGRP LE*	Unigene46613	XP_005187371.1	100%	0.0	99%	Peptidoglycan-recognition protein LE-like
*MdPGRP SC*	CL4993.Contig2	XP_005186585.1	83%	2e-111	81%	Peptidoglycan-recognition protein SC2-like isoform X1
*MdproPO 1*	CL572.Contig6	XP_005179890.1	93%	0.0	84%	Phenoloxidase subunit A3-like
*MdPAP 1*	CL4801.Contig2	XP_005176689.1	100%	0.0	99%	Serine protease easter-like
*MdPAP 2*	CL9802.Contig1	XP_005176204.1	75%	0.0	99%	Serine protease easter-like
*MdPAP 3*	CL4876.Contig1	XP_005186139.1	91%	0.0	95%	Plasminogen-like
*MdSerpin 3*	CL8948.Contig2	XP_005177808.1	99%	0.0	95%	Serine protease inhibitor 3/4-like
*MdSerpin 3*	CL5316.Contig7	XP_005177808.1	80%	9e-166	65%	Serine protease inhibitor 3/4-like
*MdSerpin 11*	Unigene14891	XP_005183089.1	94%	0.0	100%	Serpin B3-like

### Real-time RT-PCR analysis

**Figure 10:**
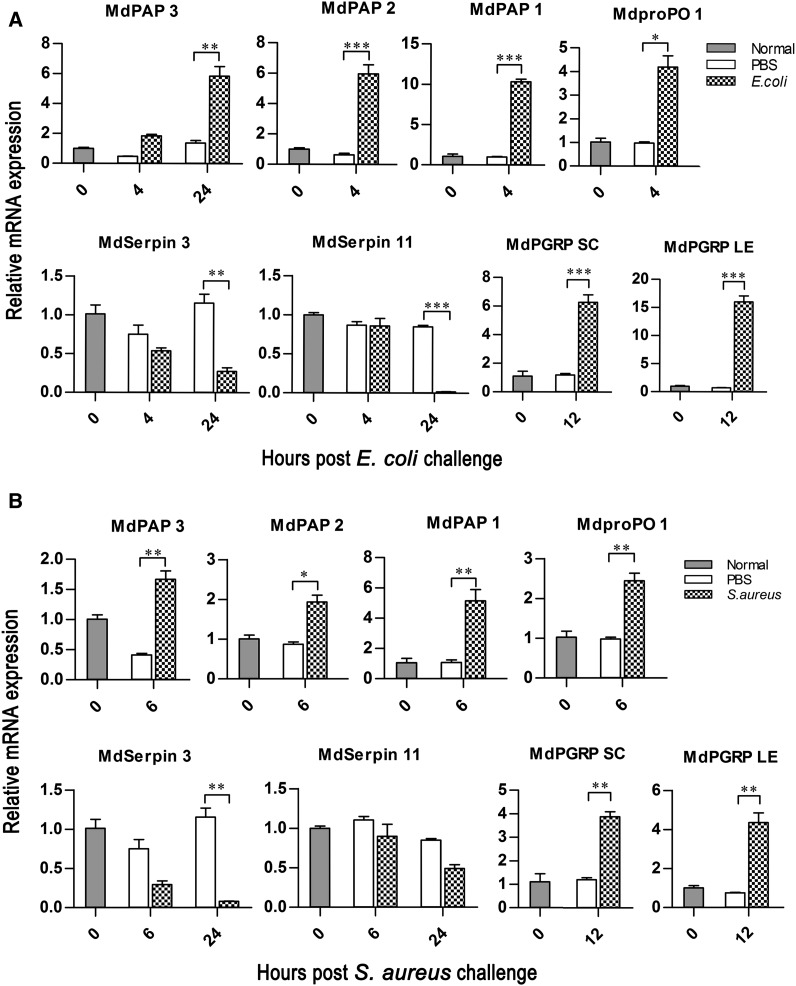
Expression profiles of candidate genes. The expression profiles of selected genes were detected by qRT-PCR using the third instar larvae at different time intervals (0, 2, 4, 6, 12, 24 hr) after challenge by *E. coli* (A) or *S. aureus* (B), in which *actin* acted as the quantity and quality control to normalize interest gene expression level. The error bars represent the mean ± SD of three repeat amplifications. The asterisks represent significant differences from the control (unpaired *t*-test, ***P < 0.001, **P < 0.01, *P < 0.05).

To verify whether the predicted genes participate in the activation of the proPO system, the expression pattern analyses were implemented on eight selected unigenes in larvae challenged by *E. coli* or *S. aureus* (at 0, 4, 6, 12, and 24 hr) using qRT-PCR methods (Figure 10). After *E. coli* infection, the significantly higher expression levels were observed at 4 hr after challenge for *mdPAP1*, *mdPAP2*, and *mdproPO1*, at 12 hr after challenge for *mdPGRP-SC* and *mdPGRP-LE*, and at 24 hr after challenge for *mdPAP3*, respectively. On the contrary, the mRNA levels of *mdSerpin3* and *mdSerpin11* decreased within 0 to 24 hr after challenge, especially at 24 hr after challenge with the most significant downregulation, as shown in Figure 10A. In comparison with *E. coli* challenge, the *S. aureus* challenge also resulted in similar expression patterns on these selected genes in larvae (Figure 10B). The similar qRT-PCR results were also observed in a previous report in which those speculated genes of *PGRP*, *PAP*, and *proPO* were all upregulated except *Serpin*, whose expression pattern was strangely upregulated in *M. domestica* larvae (Tang *et al.* 2014). In the present study, the expression profiles of the tested genes were mainly inconsistent with the activating progress of proPO system, which may reflect their functional diversifications in the activation of the proPO system in *M. domestica*.

### Measurement of phenoloxidase activity

**Figure 11:**
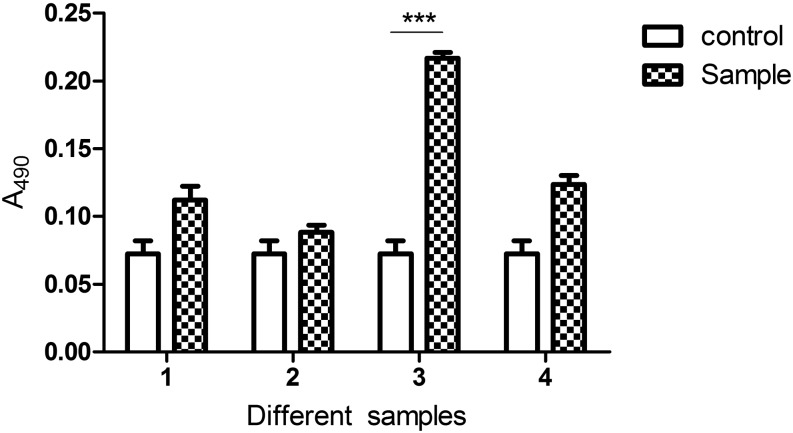
Measurement of PO activity. Phenoloxidase (PO) activity of hemolymph samples were assayed with 96-well microplates method using L-DOPA as a substrate. The PO level in hemolymph from normal larvae remained constant, as marked in the control (blank column). Compared with controls, four sample groups marked 1, 2, 3, and 4 (decorative columns) showed the variable increases in PO activity in the hemolymph. The most significant increase in PO activity appeared in sample 3 from larvae challenged by *S. aureus* and *E. coli* for 30 min. The lowest increase in PO activity existed in sample 2 from larvae injected with mdproPO 1 antibodies. Importantly, the marked increase in PO activity in bacteria-infected larvae was reverse of the quietly low level after blocking with mdproPO 1 antibodies in sample 4, which was close to the PO level in sample 1 from PBS challenged larvae. The significant variation between control and tested samples was calculated by *t*-test (***P < 0.001, **P < 0.01, *P < 0.05).

We had previously measured PO activity that could increase rapidly in housefly larvae after challenge by *S. aureus* and *E. coli* with the 96-well microplates method (Guo *et al.* 2014) but did not know whether it was related to mdproPO1 cleavage into mdPO1. Here, prior to immune challenge by the mixture of *S. aureus* and *E. coli*, housefly larvae were injected with mdproPO1 antibodies to block the interior mdproPO1 proteins. As an important result, the PO activity increase was reversed to the very low level in infected larvae after blocking with mdproPO1 antibodies (Figure 11). It was confirmed that mdproPO1 can play an indispensable role in the activation of proPO system in *M. domestica*.

### Conclusion

We have constructed the combined transcriptome by Illumina Hiseq2000 sequencing using multiple RNA samples from normal and bacteria-challenged larvae, pupae, and adults of *M. domestica*. The 4.93-Gb nucleotides were obtained and orderly assembled into 54.8 million clean reads, 223,936 contigs, and 89,842 unigenes, which represented the comprehensive transcriptomic resource currently available in *M. domestica*.

This study is the first to identify 24 putative genes related to the housefly proPO system, including eight *mdPGRP*, two *mdproPO*, three *mdPAP*, and 11 *mdSerpin*, but no *mdSPH*. The sequence alignment has demonstrated that these putative genes were highly reliable to their matched sequences in *M. domestica* genome. The qRT-PCR results also suggested that these putative genes might participate in the activating process of the housefly proPO system. It is important that the activation of the proPO system has been testified by measuring the changes of PO activity in bacteria-infected larvae after proPO antibody blockage.

Conclusively, this work may serve as a substantial foundation to outline the framework of the proPO system and further study the activation mechanism and immune defense functions of the proPO system in *M. domestica*.

## Supplementary Material

Supporting Information
