# Supplementary material for: Transcriptomic Analysis of Musca domestica to Reveal Key Genes of the Prophenoloxidase-Activating System
Source: G3 (Bethesda). 2015 Jul 7;5(9):1827–41. doi: 10.1534/g3.115.016899 (PMC4555219; doi:10.1534/g3.115.016899)
Supplement: Supporting Information [file supp_5_9_1827__index.html]

Transcriptomic Analysis of Musca domestica to Reveal Key Genes of the Prophenoloxidase-Activating System — Supporting Information 

# Transcriptomic Analysis of *Musca domestica* to Reveal Key Genes of the Prophenoloxidase-Activating System

## Supporting Information for Li *et al.*, 2015

**Files in this Data Supplement:**

- Supporting Information - Tables S1-S2 and Figure S1 (PDF, 230 KB)
- Table S1 - Primers used in the qRT-PCR. (PDF, 168 KB)
- Table S2 - Summary of KEGG pathways. (PDF, 142 KB)
- Figure S1 - The intraspecific phylogenetic analysis of mdSerpin unigenes. (PDF, 121 KB)
