# Supplementary material for: Transcriptomic Analysis of Musca domestica to Reveal Key Genes of the Prophenoloxidase-Activating System
Source: G3 (Bethesda). 2015 Jul 7;5(9):1827–41. doi: 10.1534/g3.115.016899 (PMC4555219; doi:10.1534/g3.115.016899)
Supplement: Supporting Information [file supp_g3.115.016899_016899SI.pdf]

G3 · Genes | Genomes | Genetics

**Transcriptomic analysis of *Musca domestica* to reveal key genes of the prophenoloxidase-activating system**

Dianxiang Li[*], Yongli Liang[§], Xianwei Wang[†], Lei Wang[§, ‡], Mei Qi[*], Yang Yu[*], Yuanyuan Luan[*]

[*]Biotechnology Department, The School of Biological Sciences and Biotechnology, University of Jinan, Jinan 250022, P. R. China;

[§]Shandong Academy of Medical Sciences, Jinan 250002, P. R. China;

[†]School of Life Sciences, Shandong University, Jinan, 250100, P. R. China;

[‡]School of Medicine and Life Science, Shandong Academy of Medical Sciences, University of Jinan, Jinan 250022, P. R. China.

CORREPONDING AUTHOR

Dianxiang Li

No. 336, West Road of Nan Xinzhuang, Jinan 250022, Shandong, China

Tel: 86-531-82765807

E-mail: chm_lidx@ujn.edu.cn

**DOI: 10.1534/g3.115.016899**

**Table S1 Primers used in the qRT-PCR.**

| Putative gene | Unigene ID | Primers | Primer Seqence (5’-3’) |
|---|---|---|---|
| mdPGRP SC | CL4993.Contig2 | F | TTGGGTCTCACCATCATCTCG |
| | | R | CGCCGATCAAGAAGTTGTAGC |
| mdPGRP LE | Unigene46613 | F | TGTATGAGGGACGGGGATG |
| | | R | AATGCTCGTGGTGGTGGA |
| mdproPO 1 | Unigene74274 | F | TTATACAGTGCCCCAGTTGC |
| | | R | TGGTATAGGTGAATGGTGTATG |
| mdPAP 1 | CL4801.Contig2 | F | TGAAGTTGAAGGCAGGCGT |
| | | R | CAACCCTGGCAAACCACAT |
| MdPAP 2 | CL9802.Contig1 | F | ACAAACGATATGTCATCACTGCG |
| | | R | GGGGATGGGGTATCAGTTGC |
| mdPAP 3 | CL4876.Contig1 | F | ATTTGCTACGCCACTGGATG |
| | | R | TTCGATGCGACTCACTAACG |
| mdSerpin 3 | CL8948.Contig2 | F | TGATAATCTTGCCCAACTCTC |
| | | R | TTGTGAATAACCTTGGAGACC |
| mdSerpin 11 | Unigene14891 | F | GCACCACAACCTGTATCCT |
| | | R | CGTTGGATTTCTCCTCTTTCAGT |
| actin | CL9042.Contig2 | F | CCCTCTTCCAGCCCTCGTTC |
| | | R | CCACCGATCCAGACGGAGTA |

**Table S2 Summary of KEGG pathways.**

| No. | Pathway | Count (41578) | Pathway ID |
|---|---|---|---|
| 1 | Metabolic pathways | 5704 | ko01100 |
| 2 | Pathways in cancer | 1596 | ko05200 |
| 3 | Focal adhesion | 1469 | ko04510 |
| 4 | Regulation of actin cytoskeleton | 1289 | ko04810 |
| 5 | RNA transport | 1221 | ko03013 |
| 6 | Amoebiasis | 1216 | ko05146 |
| 7 | HTLV-I infection | 1178 | ko05166 |
| 8 | Purine metabolism | 1155 | ko00230 |
| 9 | Spliceosome | 1154 | ko03040 |
| 10 | Epstein-Barr virus infection | 1053 | ko05169 |
| 11 | Huntington's disease | 1027 | ko05016 |
| 12 | MAPK signaling pathway | 1003 | ko04010 |
| 13 | Vibrio cholerae infection | 1002 | ko05110 |
| 14 | Protein processing in endoplasmic reticulum | 990 | ko04141 |
| 15 | Endocytosis | 972 | ko04144 |
| 16 | Lysosome | 940 | ko04142 |
| 17 | Influenza A | 930 | ko05164 |
| 18 | Herpes simplex infection | 916 | ko05168 |
| 19 | Ubiquitin mediated proteolysis | 893 | ko04120 |
| 20 | Wnt signaling pathway | 880 | ko04310 |
| 21 | Transcriptional misregulation in cancer | 879 | ko05202 |
| 22 | Lysine degradation | 857 | ko00310 |
| 23 | RNA degradation | 829 | ko03018 |
| 24 | Vascular smooth muscle contraction | 802 | ko04270 |
| 25 | Dilated cardiomyopathy | 781 | ko05414 |
| 26 | Insulin signaling pathway | 775 | ko04910 |
| 27 | Phagosome | 774 | ko04145 |
| 28 | Hypertrophic cardiomyopathy (HCM) | 764 | ko05410 |
| 29 | Alzheimer's disease | 760 | ko05010 |
| 30 | mRNA surveillance pathway | 752 | ko03015 |
| 31 | Tight junction | 747 | ko04530 |
| 32 | Protein digestion and absorption | 727 | ko04974 |
| 33 | ECM-receptor interaction | 725 | ko04512 |
| 34 | Pancreatic secretion | 718 | ko04972 |
| 35 | Cell cycle | 704 | ko04110 |
| 36 | Pyrimidine metabolism | 687 | ko00240 |
| 37 | Dorso-ventral axis formation | 660 | ko04320 |
| 38 | Chemokine signaling pathway | 658 | ko04062 |
| 39 | Adherens junction | 657 | ko04520 |
| 40 | Tuberculosis | 654 | ko05152 |
| 41 | Axon guidance | 628 | ko04360 |
| 42 | Salmonella infection | 628 | ko05132 |
| 43 | Ribosome biogenesis in eukaryotes | 613 | ko03008 |
| 44 | Calcium signaling pathway | 612 | ko04020 |
| 45 | Renal cell carcinoma | 606 | ko05211 |
| 46 | Bile secretion | 574 | ko04976 |
| 47 | Salivary secretion | 534 | ko04970 |
| 48 | Oocyte meiosis | 533 | ko04114 |
| 49 | Fc gamma R-mediated phagocytosis | 528 | ko04666 |
| 50 | Amino sugar and nucleotide sugar metabolism | 523 | ko00520 |
| 51 | Dopaminergic synapse | 519 | ko04728 |
| 52 | Peroxisome | 517 | ko04146 |
| 53 | Oxidative phosphorylation | 516 | ko00190 |

| | | | |
|---|---|---|---|
| 54 | Prostate cancer | 514 | ko05215 |
| 55 | Neuroactive ligand-receptor interaction | 501 | ko04080 |
| 56 | Neurotrophin signaling pathway | 490 | ko04722 |
| 57 | Melanogenesis | 486 | ko04916 |
| 58 | Pathogenic Escherichia coli infection | 483 | ko05130 |
| 59 | Bacterial invasion of epithelial cells | 477 | ko05100 |
| 60 | Notch signaling pathway | 470 | ko04330 |
| 61 | Viral myocarditis | 467 | ko05416 |
| 62 | ABC transporters | 451 | ko02010 |
| 63 | Glycerophospholipid metabolism | 448 | ko00564 |
| 64 | Parkinson's disease | 443 | ko05012 |
| 65 | Leukocyte transendothelial migration | 432 | ko04670 |
| 66 | Long-term potentiation | 432 | ko04720 |
| 67 | T cell receptor signaling pathway | 428 | ko04660 |
| 68 | GnRH signaling pathway | 426 | ko04912 |
| 69 | TGF-beta signaling pathway | 426 | ko04350 |
| 70 | PPAR signaling pathway | 425 | ko03320 |
| 71 | Gastric acid secretion | 424 | ko04971 |
| 72 | Progesterone-mediated oocyte maturation | 423 | ko04914 |
| 73 | Phosphatidylinositol signaling system | 419 | ko04070 |
| 74 | Toxoplasmosis | 413 | ko05145 |
| 75 | Starch and sucrose metabolism | 413 | ko00500 |
| 76 | Circadian rhythm - fly | 405 | ko04711 |
| 77 | Glutamatergic synapse | 403 | ko04724 |
| 78 | Shigellosis | 402 | ko05131 |
| 79 | Jak-STAT signaling pathway | 399 | ko04630 |
| 80 | ErbB signaling pathway | 395 | ko04012 |
| 81 | Cardiac muscle contraction | 394 | ko04260 |
| 82 | RNA polymerase | 390 | ko03020 |
| 83 | Epithelial cell signaling in Helicobacter pylori infection | 389 | ko05120 |
| 84 | Glycerolipid metabolism | 370 | ko00561 |
| 85 | Small cell lung cancer | 364 | ko05222 |
| 86 | VEGF signaling pathway | 357 | ko04370 |
| 87 | Glutathione metabolism | 356 | ko00480 |
| 88 | Alcoholism | 355 | ko05034 |
| 89 | Gap junction | 353 | ko04540 |
| 90 | Inositol phosphate metabolism | 346 | ko00562 |
| 91 | Ribosome | 346 | ko03010 |
| 92 | Arrhythmogenic right ventricular cardiomyopathy (ARVC) | 346 | ko05412 |
| 93 | Amyotrophic lateral sclerosis (ALS) | 343 | ko05014 |
| 94 | Drug metabolism - other enzymes | 338 | ko00983 |
| 95 | Basal transcription factors | 334 | ko03022 |
| 96 | Synaptic vesicle cycle | 326 | ko04721 |
| 97 | mTOR signaling pathway | 323 | ko04150 |
| 98 | Aminoacyl-tRNA biosynthesis | 323 | ko00970 |
| 99 | MAPK signaling pathway - fly | 309 | ko04013 |
| 100 | Hepatitis C | 305 | ko05160 |
| 101 | Natural killer cell mediated cytotoxicity | 297 | ko04650 |
| 102 | Hedgehog signaling pathway | 297 | ko04340 |
| 103 | Measles | 296 | ko05162 |
| 104 | Fatty acid metabolism | 295 | ko00071 |
| 105 | Vasopressin-regulated water reabsorption | 295 | ko04962 |
| 106 | Cholinergic synapse | 291 | ko04725 |
| 107 | Osteoclast differentiation | 291 | ko04380 |

| | | | |
|---|---|---|---|
| 108 | B cell receptor signaling pathway | 290 | ko04662 |
| 109 | Pentose and glucuronate interconversions | 277 | ko00040 |
| 110 | Adipocytokine signaling pathway | 277 | ko04920 |
| 111 | Chronic myeloid leukemia | 272 | ko05220 |
| 112 | Chagas disease (American trypanosomiasis) | 270 | ko05142 |
| 113 | Fc epsilon RI signaling pathway | 269 | ko04664 |
| 114 | Endometrial cancer | 269 | ko05213 |
| 115 | Vitamin digestion and absorption | 265 | ko04977 |
| 116 | Morphine addiction | 265 | ko05032 |
| 117 | Valine, leucine and isoleucine degradation | 264 | ko00280 |
| 118 | Cell adhesion molecules (CAMs) | 263 | ko04514 |
| 119 | Arginine and proline metabolism | 260 | ko00330 |
| 120 | Fat digestion and absorption | 260 | ko04975 |
| 121 | Prion diseases | 260 | ko05020 |
| 122 | Glycine, serine and threonine metabolism | 255 | ko00260 |
| 123 | Basal cell carcinoma | 254 | ko05217 |
| 124 | Pyruvate metabolism | 249 | ko00620 |
| 125 | Colorectal cancer | 248 | ko05210 |
| 126 | Glycolysis / Gluconeogenesis | 248 | ko00010 |
| 127 | Galactose metabolism | 246 | ko00052 |
| 128 | GABAergic synapse | 242 | ko04727 |
| 129 | Drug metabolism - cytochrome P450 | 241 | ko00982 |
| 130 | Citrate cycle (TCA cycle) | 239 | ko00020 |
| 131 | Retrograde endocannabinoid signaling | 238 | ko04723 |
| 132 | N-Glycan biosynthesis | 238 | ko00510 |
| 133 | Metabolism of xenobiotics by cytochrome P450 | 237 | ko00980 |
| 134 | Serotonergic synapse | 237 | ko04726 |
| 135 | Cytosolic DNA-sensing pathway | 228 | ko04623 |
| 136 | Nucleotide excision repair | 228 | ko03420 |
| 137 | Glioma | 228 | ko05214 |
| 138 | Toll-like receptor signaling pathway | 224 | ko04620 |
| 139 | Legionellosis | 222 | ko05134 |
| 140 | Rheumatoid arthritis | 222 | ko05323 |
| 141 | Tyrosine metabolism | 222 | ko00350 |
| 142 | Hematopoietic cell lineage | 221 | ko04640 |
| 143 | Endocrine and other factor-regulated calcium reabsorption | 214 | ko04961 |
| 144 | alpha-Linolenic acid metabolism | 213 | ko00592 |
| 145 | Cysteine and methionine metabolism | 213 | ko00270 |
| 146 | Mineral absorption | 212 | ko04978 |
| 147 | Complement and coagulation cascades | 209 | ko04610 |
| 148 | Propanoate metabolism | 208 | ko00640 |
| 149 | Amphetamine addiction | 205 | ko05031 |
| 150 | Porphyrin and chlorophyll metabolism | 202 | ko00860 |
| 151 | Renin-angiotensin system | 200 | ko04614 |
| 152 | Pancreatic cancer | 199 | ko05212 |
| 153 | Alanine, aspartate and glutamate metabolism | 199 | ko00250 |
| 154 | Retinol metabolism | 195 | ko00830 |
| 155 | p53 signaling pathway | 194 | ko04115 |
| 156 | Carbohydrate digestion and absorption | 191 | ko04973 |
| 157 | Apoptosis | 191 | ko04210 |
| 158 | Non-small cell lung cancer | 190 | ko05223 |
| 159 | Long-term depression | 187 | ko04730 |
| 160 | DNA replication | 186 | ko03030 |
| 161 | beta-Alanine metabolism | 183 | ko00410 |
| 162 | Proteasome | 181 | ko03050 |

| | | | |
|---|---|---|---|
| 163 | Olfactory transduction | 179 | ko04740 |
| 164 | Sphingolipid metabolism | 178 | ko00600 |
| 165 | Phototransduction - fly | 176 | ko04745 |
| 166 | Circadian rhythm - mammal | 175 | ko04710 |
| 167 | Ascorbate and aldarate metabolism | 175 | ko00053 |
| 168 | Tryptophan metabolism | 172 | ko00380 |
| 169 | Acute myeloid leukemia | 170 | ko05221 |
| 170 | Fanconi anemia pathway | 168 | ko03460 |
| 171 | Collecting duct acid secretion | 164 | ko04966 |
| 172 | Pentose phosphate pathway | 164 | ko00030 |
| 173 | Fructose and mannose metabolism | 163 | ko00051 |
| 174 | Pertussis | 161 | ko05133 |
| 175 | Biosynthesis of unsaturated fatty acids | 161 | ko01040 |
| 176 | NF-kappa B signaling pathway | 159 | ko04064 |
| 177 | Other types of O-glycan biosynthesis | 152 | ko00514 |
| 178 | Malaria | 151 | ko05144 |
| 179 | Antigen processing and presentation | 147 | ko04612 |
| 180 | Base excision repair | 145 | ko03410 |
| 181 | Type II diabetes mellitus | 144 | ko04930 |
| 182 | Cytokine-cytokine receptor interaction | 143 | ko04060 |
| 183 | Folate biosynthesis | 142 | ko00790 |
| 184 | Aldosterone-regulated sodium reabsorption | 142 | ko04960 |
| 185 | Thyroid cancer | 137 | ko05216 |
| 186 | Glycosylphosphatidylinositol(GPI)-anchor biosynthesis | 136 | ko00563 |
| 187 | Butanoate metabolism | 136 | ko00650 |
| 188 | Other glycan degradation | 125 | ko00511 |
| 189 | Glyoxylate and dicarboxylate metabolism | 124 | ko00630 |
| 190 | Steroid hormone biosynthesis | 123 | ko00140 |
| 191 | Ether lipid metabolism | 123 | ko00565 |
| 192 | Mismatch repair | 122 | ko03430 |
| 193 | Melanoma | 121 | ko05218 |
| 194 | Proximal tubule bicarbonate reclamation | 121 | ko04964 |
| 195 | Fatty acid elongation | 120 | ko00062 |
| 196 | Homologous recombination | 119 | ko03440 |
| 197 | Insect hormone biosynthesis | 118 | ko00981 |
| 198 | Staphylococcus aureus infection | 116 | ko05150 |
| 199 | Systemic lupus erythematosus | 115 | ko05322 |
| 200 | Protein export | 115 | ko03060 |
| 201 | Glycosaminoglycan degradation | 115 | ko00531 |
| 202 | Phenylalanine metabolism | 113 | ko00360 |
| 203 | Bladder cancer | 113 | ko05219 |
| 204 | Phototransduction | 111 | ko04744 |
| 205 | SNARE interactions in vesicular transport | 109 | ko04130 |
| 206 | NOD-like receptor signaling pathway | 108 | ko04621 |
| 207 | RIG-I-like receptor signaling pathway | 107 | ko04622 |
| 208 | Leishmaniasis | 104 | ko05140 |
| 209 | Glycosaminoglycan biosynthesis - heparan sulfate | 102 | ko00534 |
| 210 | Riboflavin metabolism | 101 | ko00740 |
| 211 | Cocaine addiction | 101 | ko05030 |
| 212 | Nicotinate and nicotinamide metabolism | 96 | ko00760 |
| 213 | Regulation of autophagy | 94 | ko04140 |
| 214 | Terpenoid backbone biosynthesis | 93 | ko00900 |
| 215 | One carbon pool by folate | 92 | ko00670 |
| 216 | Arachidonic acid metabolism | 91 | ko00590 |
| 217 | Maturity onset diabetes of the young | 86 | ko04950 |

| | | | |
|---|---|---|---|
| 218 | Fatty acid biosynthesis | 80 | ko00061 |
| 219 | Mucin type O-Glycan biosynthesis | 79 | ko00512 |
| 220 | Taste transduction | 70 | ko04742 |
| 221 | Pantothenate and CoA biosynthesis | 68 | ko00770 |
| 222 | Histidine metabolism | 67 | ko00340 |
| 223 | African trypanosomiasis | 59 | ko05143 |
| 224 | Primary immunodeficiency | 58 | ko05340 |
| 225 | Steroid biosynthesis | 58 | ko00100 |
| 226 | Selenocompound metabolism | 56 | ko00450 |
| 227 | Linoleic acid metabolism | 54 | ko00591 |
| 228 | Glycosphingolipid biosynthesis - ganglio series | 53 | ko00604 |
| 229 | Glycosaminoglycan biosynthesis - chondroitin sulfate | 52 | ko00532 |
| 230 | Caffeine metabolism | 50 | ko00232 |
| 231 | Ubiquinone and other terpenoid-quinone biosynthesis | 49 | ko00130 |
| 232 | Cyanoamino acid metabolism | 49 | ko00460 |
| 233 | Non-homologous end-joining | 47 | ko03450 |
| 234 | Nicotine addiction | 44 | ko05033 |
| 235 | Glycosphingolipid biosynthesis - globo series | 44 | ko00603 |
| 236 | Sulfur relay system | 42 | ko04122 |
| 237 | Primary bile acid biosynthesis | 40 | ko00120 |
| 238 | Synthesis and degradation of ketone bodies | 40 | ko00072 |
| 239 | Sulfur metabolism | 36 | ko00920 |
| 240 | Autoimmune thyroid disease | 35 | ko05320 |
| 241 | Valine, leucine and isoleucine biosynthesis | 31 | ko00290 |
| 242 | Glycosaminoglycan biosynthesis - keratan sulfate | 30 | ko00533 |
| 243 | Glycosphingolipid biosynthesis - lacto and neolacto series | 27 | ko00601 |
| 244 | Type I diabetes mellitus | 27 | ko04940 |
| 245 | Taurine and hypotaurine metabolism | 27 | ko00430 |
| 246 | Phenylalanine, tyrosine and tryptophan biosynthesis | 25 | ko00400 |
| 247 | Lipoic acid metabolism | 21 | ko00785 |
| 248 | D-Glutamine and D-glutamate metabolism | 20 | ko00471 |
| 249 | Vitamin B6 metabolism | 20 | ko00750 |
| 250 | D-Arginine and D-ornithine metabolism | 18 | ko00472 |
| 251 | Biotin metabolism | 15 | ko00780 |
| 252 | Butirosin and neomycin biosynthesis | 13 | ko00524 |
| 253 | Asthma | 9 | ko05310 |
| 254 | Lysine biosynthesis | 8 | ko00300 |
| 255 | Thiamine metabolism | 6 | ko00730 |
| 256 | Allograft rejection | 3 | ko05330 |
| 257 | Graft-versus-host disease | 3 | ko05332 |
| 258 | Intestinal immune network for IgA production | 3 | ko04672 |

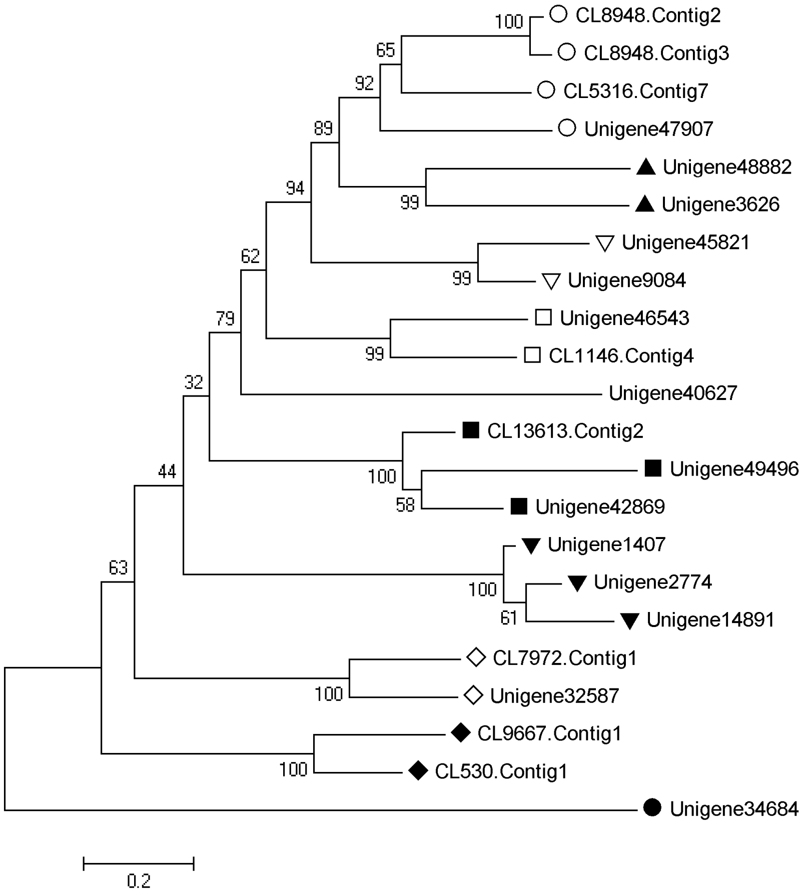


**Figure S1 The intraspecific phylogenetic analysis of mdSerpin unigenes.** The amino acid sequences from 22 mdSerpin unigenes of *M. domestica* were used to build the NJ phylogenetic tree by MEGA 5.0 with 1000 bootstraps. The unigenes were clustered different groups with different marks.
