# Supplementary material for: Transcriptomic Analysis of Musca domestica to Reveal Key Genes of the Prophenoloxidase-Activating System
Source: G3 (Bethesda). 2015 Jul 7;5(9):1827–41. doi: 10.1534/g3.115.016899 (PMC4555219; doi:10.1534/g3.115.016899)
Supplement: Supporting Information [file supp_g3.115.016899_TableS1.pdf]

Table S1 Primers used in the qRT-PCR.

Putative gene	Unigene ID	Primers	Primer Seqence (5'-3')
mdPGRP SC	CL4993.Contig2	F	TTGGGTCTCACCATCATCTCG
		R	CGCCGATCAAGAAGTTGTAGC
mdPGRP LE	Unigene46613	F	TGTATGAGGGACGGGGATG
		R	AATGCTCGTGGTGGTGGA
mdproPO 1	Unigene74274	F	TTATACAGTGCCCCAGTTGC
		R	TGGTATAGGTGAATGGTGTATG
mdPAP 1	CL4801.Contig2	F	TGAAGTTGAAGGCAGGCGT
		R	CAACCCTGGCAAACCACAT
MdPAP 2	CL9802.Contig1	F	ACAAACGATATGTCATCACTGCG
		R	GGGGATGGGGTATCAGTTGC
mdPAP 3	CL4876.Contig1	F	ATTTGCTACGCCACTGGATG
		R	TTCGATGCGACTCACTAACG
mdSerpin 3	CL8948.Contig2	F	TGATAATCTTGCCCAACTCTC
		R	TTGTGAATAACCTTGGAGACC
mdSerpin 11	Unigene14891	F	GCACCACAACCTGTATCCT
		R	CGTTGGATTTCTCCTCTTTCAGT
actin	CL9042.Contig2	F	CCCTCTTCCAGCCCTCGTTC
		R	CCACCGATCCAGACGGAGTA
