# Supplementary material for: Transcriptomic Analysis of Musca domestica to Reveal Key Genes of the Prophenoloxidase-Activating System
Source: G3 (Bethesda). 2015 Jul 7;5(9):1827–41. doi: 10.1534/g3.115.016899 (PMC4555219; doi:10.1534/g3.115.016899)
Supplement: Supporting Information [file supp_g3.115.016899_TableS2.pdf]

Table S2 Summary of KEGG pathways.

No.	Pathway	Count (41578)	Pathway ID
1	Metabolic pathways	5704	ko01100
2	Pathways in cancer	1596	ko05200
3	Focal adhesion	1469	ko04510
4	Regulation of actin cytoskeleton	1289	ko04810
5	RNA transport	1221	ko03013
6	Amoebiasis	1216	ko05146
7	HTLV-I infection	1178	ko05166
8	Purine metabolism	1155	ko00230
9	Spliceosome	1154	ko03040
10	Epstein-Barr virus infection	1053	ko05169
11	Huntington's disease	1027	ko05016
12	MAPK signaling pathway	1003	ko04010
13	Vibrio cholerae infection	1002	ko05110
14	Protein processing in endoplasmic reticulum	990	ko04141
15	Endocytosis	972	ko04144
16	Lysosome	940	ko04142
17	Influenza A	930	ko05164
18	Herpes simplex infection	916	ko05168
19	Ubiquitin mediated proteolysis	893	ko04120
20	Wnt signaling pathway	880	ko04310
21	Transcriptional misregulation in cancer	879	ko05202
22	Lysine degradation	857	ko00310
23	RNA degradation	829	ko03018
24	Vascular smooth muscle contraction	802	ko04270
25	Dilated cardiomyopathy	781	ko05414
26	Insulin signaling pathway	775	ko04910
27	Phagosome	774	ko04145
28	Hypertrophic cardiomyopathy (HCM)	764	ko05410
29	Alzheimer's disease	760	ko05010
30	mRNA surveillance pathway	752	ko03015
31	Tight junction	747	ko04530
32	Protein digestion and absorption	727	ko04974
33	ECM-receptor interaction	725	ko04512
34	Pancreatic secretion	718	ko04972
35	Cell cycle	704	ko04110
36	Pyrimidine metabolism	687	ko00240
37	Dorso-ventral axis formation	660	ko04320
38	Chemokine signaling pathway	658	ko04062
39	Adherens junction	657	ko04520
40	Tuberculosis	654	ko05152
41	Axon guidance	628	ko04360
42	Salmonella infection	628	ko05132
43	Ribosome biogenesis in eukaryotes	613	ko03008
44	Calcium signaling pathway	612	ko04020
45	Renal cell carcinoma	606	ko05211
46	Bile secretion	574	ko04976
47	Salivary secretion	534	ko04970
48	Oocyte meiosis	533	ko04114
49	Fc gamma R-mediated phagocytosis	528	ko04666
50	Amino sugar and nucleotide sugar metabolism	523	ko00520
51	Dopaminergic synapse	519	ko04728
52	Peroxisome	517	ko04146
53	Oxidative phosphorylation	516	ko00190

54	Prostate cancer	514	ko05215
55	Neuroactive ligand-receptor interaction	501	ko04080
56	Neurotrophin signaling pathway	490	ko04722
57	Melanogenesis	486	ko04916
58	Pathogenic Escherichia coli infection	483	ko05130
59	Bacterial invasion of epithelial cells	477	ko05100
60	Notch signaling pathway	470	ko04330
61	Viral myocarditis	467	ko05416
62	ABC transporters	451	ko02010
63	Glycerophospholipid metabolism	448	ko00564
64	Parkinson's disease	443	ko05012
65	Leukocyte transendothelial migration	432	ko04670
66	Long-term potentiation	432	ko04720
67	T cell receptor signaling pathway	428	ko04660
68	GnRH signaling pathway	426	ko04912
69	TGF-beta signaling pathway	426	ko04350
70	PPAR signaling pathway	425	ko03320
71	Gastric acid secretion	424	ko04971
72	Progesterone-mediated oocyte maturation	423	ko04914
73	Phosphatidylinositol signaling system	419	ko04070
74	Toxoplasmosis	413	ko05145
75	Starch and sucrose metabolism	413	ko00500
76	Circadian rhythm - fly	405	ko04711
77	Glutamatergic synapse	403	ko04724
78	Shigellosis	402	ko05131
79	Jak-STAT signaling pathway	399	ko04630
80	ErbB signaling pathway	395	ko04012
81	Cardiac muscle contraction	394	ko04260
82	RNA polymerase	390	ko03020
83	Epithelial cell signaling in Helicobacter pylori infection	389	ko05120
84	Glycerolipid metabolism	370	ko00561
85	Small cell lung cancer	364	ko05222
86	VEGF signaling pathway	357	ko04370
87	Glutathione metabolism	356	ko00480
88	Alcoholism	355	ko05034
89	Gap junction	353	ko04540
90	Inositol phosphate metabolism	346	ko00562
91	Ribosome	346	ko03010
92	Arrhythmogenic right ventricular cardiomyopathy (ARVC)	346	ko05412
93	Amyotrophic lateral sclerosis (ALS)	343	ko05014
94	Drug metabolism - other enzymes	338	ko00983
95	Basal transcription factors	334	ko03022
96	Synaptic vesicle cycle	326	ko04721
97	mTOR signaling pathway	323	ko04150
98	Aminoacyl-tRNA biosynthesis	323	ko00970
99	MAPK signaling pathway - fly	309	ko04013
100	Hepatitis C	305	ko05160
101	Natural killer cell mediated cytotoxicity	297	ko04650
102	Hedgehog signaling pathway	297	ko04340
103	Measles	296	ko05162
104	Fatty acid metabolism	295	ko00071
105	Vasopressin-regulated water reabsorption	295	ko04962
106	Cholinergic synapse	291	ko04725
107	Osteoclast differentiation	291	ko04380

108	B cell receptor signaling pathway	290	ko04662
109	Pentose and glucuronate interconversions	277	ko00040
110	Adipocytokine signaling pathway	277	ko04920
111	Chronic myeloid leukemia	272	ko05220
112	Chagas disease (American trypanosomiasis)	270	ko05142
113	Fc epsilon RI signaling pathway	269	ko04664
114	Endometrial cancer	269	ko05213
115	Vitamin digestion and absorption	265	ko04977
116	Morphine addiction	265	ko05032
117	Valine, leucine and isoleucine degradation	264	ko00280
118	Cell adhesion molecules (CAMs)	263	ko04514
119	Arginine and proline metabolism	260	ko00330
120	Fat digestion and absorption	260	ko04975
121	Prion diseases	260	ko05020
122	Glycine, serine and threonine metabolism	255	ko00260
123	Basal cell carcinoma	254	ko05217
124	Pyruvate metabolism	249	ko00620
125	Colorectal cancer	248	ko05210
126	Glycolysis / Gluconeogenesis	248	ko00010
127	Galactose metabolism	246	ko00052
128	GABAergic synapse	242	ko04727
129	Drug metabolism - cytochrome P450	241	ko00982
130	Citrate cycle (TCA cycle)	239	ko00020
131	Retrograde endocannabinoid signaling	238	ko04723
132	N-Glycan biosynthesis	238	ko00510
133	Metabolism of xenobiotics by cytochrome P450	237	ko00980
134	Serotonergic synapse	237	ko04726
135	Cytosolic DNA-sensing pathway	228	ko04623
136	Nucleotide excision repair	228	ko03420
137	Glioma	228	ko05214
138	Toll-like receptor signaling pathway	224	ko04620
139	Legionellosis	222	ko05134
140	Rheumatoid arthritis	222	ko05323
141	Tyrosine metabolism	222	ko00350
142	Hematopoietic cell lineage	221	ko04640
143	Endocrine and other factor-regulated calcium reabsorption	214	ko04961
144	alpha-Linolenic acid metabolism	213	ko00592
145	Cysteine and methionine metabolism	213	ko00270
146	Mineral absorption	212	ko04978
147	Complement and coagulation cascades	209	ko04610
148	Propanoate metabolism	208	ko00640
149	Amphetamine addiction	205	ko05031
150	Porphyrin and chlorophyll metabolism	202	ko00860
151	Renin-angiotensin system	200	ko04614
152	Pancreatic cancer	199	ko05212
153	Alanine, aspartate and glutamate metabolism	199	ko00250
154	Retinol metabolism	195	ko00830
155	p53 signaling pathway	194	ko04115
156	Carbohydrate digestion and absorption	191	ko04973
157	Apoptosis	191	ko04210
158	Non-small cell lung cancer	190	ko05223
159	Long-term depression	187	ko04730
160	DNA replication	186	ko03030
161	beta-Alanine metabolism	183	ko00410
162	Proteasome	181	ko03050

163	Olfactory transduction	179	ko04740
164	Sphingolipid metabolism	178	ko00600
165	Phototransduction - fly	176	ko04745
166	Circadian rhythm - mammal	175	ko04710
167	Ascorbate and aldarate metabolism	175	ko00053
168	Tryptophan metabolism	172	ko00380
169	Acute myeloid leukemia	170	ko05221
170	Fanconi anemia pathway	168	ko03460
171	Collecting duct acid secretion	164	ko04966
172	Pentose phosphate pathway	164	ko00030
173	Fructose and mannose metabolism	163	ko00051
174	Pertussis	161	ko05133
175	Biosynthesis of unsaturated fatty acids	161	ko01040
176	NF-kappa B signaling pathway	159	ko04064
177	Other types of O-glycan biosynthesis	152	ko00514
178	Malaria	151	ko05144
179	Antigen processing and presentation	147	ko04612
180	Base excision repair	145	ko03410
181	Type II diabetes mellitus	144	ko04930
182	Cytokine-cytokine receptor interaction	143	ko04060
183	Folate biosynthesis	142	ko00790
184	Aldosterone-regulated sodium reabsorption	142	ko04960
185	Thyroid cancer	137	ko05216
186	Glycosylphosphatidylinositol(GPI)-anchor biosynthesis	136	ko00563
187	Butanoate metabolism	136	ko00650
188	Other glycan degradation	125	ko00511
189	Glyoxylate and dicarboxylate metabolism	124	ko00630
190	Steroid hormone biosynthesis	123	ko00140
191	Ether lipid metabolism	123	ko00565
192	Mismatch repair	122	ko03430
193	Melanoma	121	ko05218
194	Proximal tubule bicarbonate reclamation	121	ko04964
195	Fatty acid elongation	120	ko00062
196	Homologous recombination	119	ko03440
197	Insect hormone biosynthesis	118	ko00981
198	Staphylococcus aureus infection	116	ko05150
199	Systemic lupus erythematosus	115	ko05322
200	Protein export	115	ko03060
201	Glycosaminoglycan degradation	115	ko00531
202	Phenylalanine metabolism	113	ko00360
203	Bladder cancer	113	ko05219
204	Phototransduction	111	ko04744
205	SNARE interactions in vesicular transport	109	ko04130
206	NOD-like receptor signaling pathway	108	ko04621
207	RIG-I-like receptor signaling pathway	107	ko04622
208	Leishmaniasis	104	ko05140
209	Glycosaminoglycan biosynthesis - heparan sulfate	102	ko00534
210	Riboflavin metabolism	101	ko00740
211	Cocaine addiction	101	ko05030
212	Nicotinate and nicotinamide metabolism	96	ko00760
213	Regulation of autophagy	94	ko04140
214	Terpenoid backbone biosynthesis	93	ko00900
215	One carbon pool by folate	92	ko00670
216	Arachidonic acid metabolism	91	ko00590
217	Maturity onset diabetes of the young	86	ko04950

218	Fatty acid biosynthesis	80	ko00061
219	Mucin type O-Glycan biosynthesis	79	ko00512
220	Taste transduction	70	ko04742
221	Pantothenate and CoA biosynthesis	68	ko00770
222	Histidine metabolism	67	ko00340
223	African trypanosomiasis	59	ko05143
224	Primary immunodeficiency	58	ko05340
225	Steroid biosynthesis	58	ko00100
226	Selenocompound metabolism	56	ko00450
227	Linoleic acid metabolism	54	ko00591
228	Glycosphingolipid biosynthesis - ganglio series	53	ko00604
229	Glycosaminoglycan biosynthesis - chondroitin sulfate	52	ko00532
230	Caffeine metabolism	50	ko00232
231	Ubiquinone and other terpenoid-quinone biosynthesis	49	ko00130
232	Cyanoamino acid metabolism	49	ko00460
233	Non-homologous end-joining	47	ko03450
234	Nicotine addiction	44	ko05033
235	Glycosphingolipid biosynthesis - globo series	44	ko00603
236	Sulfur relay system	42	ko04122
237	Primary bile acid biosynthesis	40	ko00120
238	Synthesis and degradation of ketone bodies	40	ko00072
239	Sulfur metabolism	36	ko00920
240	Autoimmune thyroid disease	35	ko05320
241	Valine, leucine and isoleucine biosynthesis	31	ko00290
242	Glycosaminoglycan biosynthesis - keratan sulfate	30	ko00533
243	Glycosphingolipid biosynthesis - lacto and neolacto series	27	ko00601
244	Type I diabetes mellitus	27	ko04940
245	Taurine and hypotaurine metabolism	27	ko00430
246	Phenylalanine, tyrosine and tryptophan biosynthesis	25	ko00400
247	Lipoic acid metabolism	21	ko00785
248	D-Glutamine and D-glutamate metabolism	20	ko00471
249	Vitamin B6 metabolism	20	ko00750
250	D-Arginine and D-ornithine metabolism	18	ko00472
251	Biotin metabolism	15	ko00780
252	Butirosin and neomycin biosynthesis	13	ko00524
253	Asthma	9	ko05310
254	Lysine biosynthesis	8	ko00300
255	Thiamine metabolism	6	ko00730
256	Allograft rejection	3	ko05330
257	Graft-versus-host disease	3	ko05332
258	Intestinal immune network for IgA production	3	ko04672
