# Supplementary material for: Transcriptomic Analysis of Musca domestica to Reveal Key Genes of the Prophenoloxidase-Activating System
Source: G3 (Bethesda). 2015 Jul 7;5(9):1827–41. doi: 10.1534/g3.115.016899 (PMC4555219; doi:10.1534/g3.115.016899)
Supplement: Supporting Information [file supp_g3.115.016899_FigureS1.pdf]

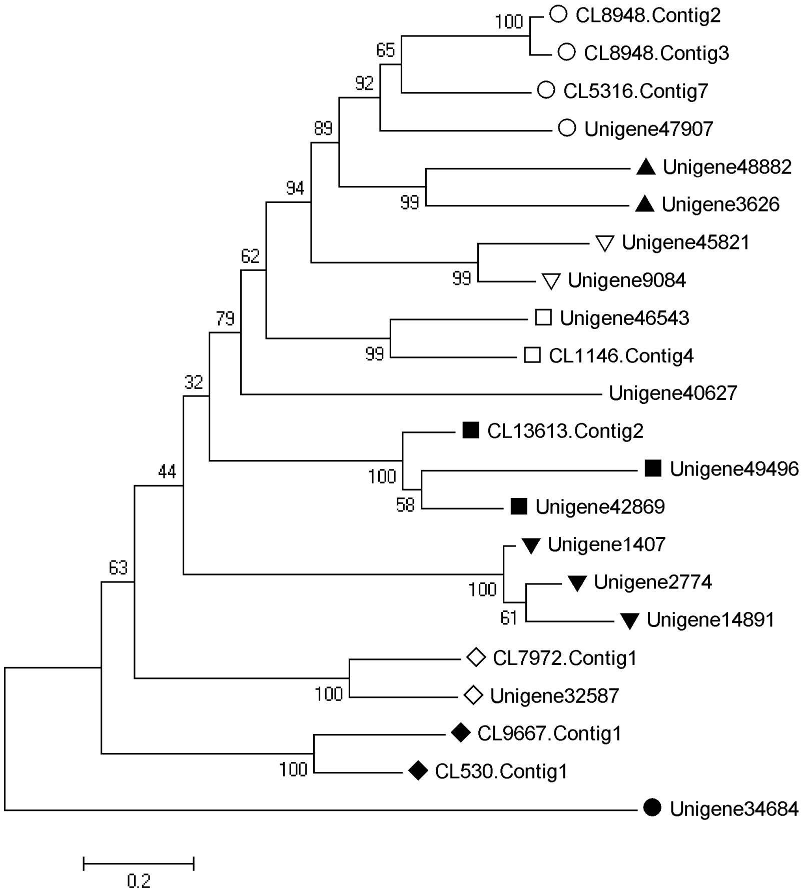

Figure S1 The intraspecific phylogenetic analysis of mdSerpin unigenes. The amino acid sequences from 22 mdSerpin unigenes of *M. domestica* were used to build the NJ phylogenetic tree by MEGA 5.0 with 1000 bootstraps. The unigenes were clustered different groups with different marks.
